# Artificial Intelligence-Powered Raman Spectroscopy through Open Science and FAIR Principles

**DOI:** 10.1021/acsnano.5c09165

**Published:** 2025-10-27

**Authors:** Nicolas Coca-Lopez, Victor Alcolea-Rodriguez, Miguel A. Bañares, Sandor Brockhauser, Julien Gorenflot, Alex Henderson, Ron Hildebrandt, Nina Jeliazkova, Nikolay Kochev, Enrique Lozano Diz, Zdenek Pilat, Dario Polli, Philip Strömert, Chris Sturm, Renzo Vanna, Raquel Portela

**Affiliations:** † 83076 Instituto de Catalisis y Petroleoquimica (ICP), CSIC, Madrid 28049, Spain; ‡ CNR-Institute for Photonics and Nanotechnologies (CNR-IFN), Piazza Leonardo Da Vinci 32, Milan 20133, Italy; § 9373 Humboldt-Universität zu Berlin, Berlin 12489, Germany; ∥ Physical Sciences and Engineering Division (PSE), 127355 King Abdullah University of Science and Technology (KAUST), Thuwal 23955-6900, Kingdom of Saudi Arabia; ⊥ Manchester Institute of Biotechnology, 5292 The University of Manchester, Manchester M1 7DN, U.K.; # 28359 German National Library of Science and Technology (TIB)—Leibniz Information Centre for Science and Technology—University Library, Hannover 30167, Germany; ¶ Ideaconsult Ltd., Sofia 1000, Bulgaria; ∇ Department of Analytical Chemistry and Computer Chemistry, University Plovdiv, 24 Tsar Asen Str., Plovdiv 4000, Bulgaria; ○ ELODIZ Ltd, High Wycombe HP11 2LT Bucks, U.K.; ⧫ Institute of Scientific Instruments of the Czech Academy of Sciences, Kralovopolska 147, Brno 612 64, Czech Republic; †† Department of Physics, Politecnico di Milano, P.zza Leonardo da Vinci 32, Milan 20133, Italy; ‡‡ Felix Bloch Institute for Solid State Physics, University Leipzig, Lepzig D04103, Germany

**Keywords:** digitalization, chemometrics, open source, hardware, software, databases, data structure, machine learning, FAIR principles, standards

## Abstract

Raman spectroscopy is a fast-growing and increasingly powerful analytical technique applied across diverse disciplines such as materials science, chemistry, biology and medicine. This growth is driven by advances in Raman instrumentation and greatly supported by the flourishing of chemometrics and artificial intelligence (AI). However, the full potential of this technique is often hampered by challenges related to data acquisition, processing, interpretation, and sharing. This review paper addresses how a concerted effort toward digitalization, incorporating principles of Open Science and FAIR data (Findable, Accessible, Interoperable, and Reusable), is essential to develop and implement robust, standardized, and accessible digital workflows. These workflows are key to unlock the full power of Raman spectroscopy in combination with AI. We explore the current landscape of digital tools and open resources in Raman spectroscopy, highlighting both existing solutions as well as critical gaps. Despite these advances, the field remains fragmented, with many initiatives developed in isolation, limiting interoperability and slowing progress. In this regard, we assess the trends in Raman spectroscopy hardware and control software as well as the role of AI in improving data collection, automating data analysis, extracting meaningful insights, and enabling predictive modeling. We review challenges such as data quality and model interpretability that constrain the effectiveness and applicability of AI in Raman spectroscopy. Furthermore, we emphasize the importance of standardized data formats, metadata schemas, and domain-specific ontologies to ensure machine-actionability, database federation and interoperability as well as to facilitate collaborative research. We provide curated lists of existing open hardware, databases and standards relevant to Raman spectroscopy. Finally, we propose a roadmap toward an open and FAIR ecosystem for Raman spectroscopy, emphasizing the need for sustainable infrastructure, collaborative development, and community involvement.

## Graphical abstract

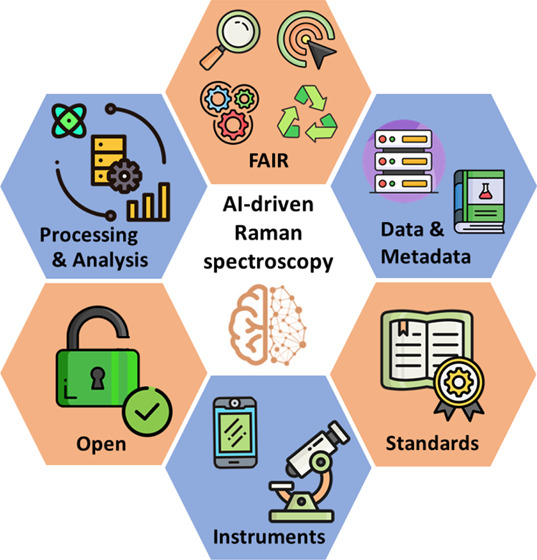

## 1 Introduction

Raman spectroscopy is a nondestructive and versatile analytical technique that provides molecular-level information through the inelastic scattering of light. By probing molecular rotational and vibrational states, Raman spectroscopy generates unique spectral fingerprints, enabling the identification and characterization of a wide range of materials. While spontaneous Raman spectroscopy provides molecular fingerprints in a straightforward manner, its inherent limitations—such as weak scattering cross-section and fluorescence interference—have driven the development of signal-enhanced techniques like Surface-Enhanced Raman Spectroscopy (SERS)^1–3^ and Tip-Enhanced Raman Spectroscopy (TERS).^4^ These approaches exploit plasmonic effects to increase the Raman signal by several orders of magnitude, significantly improving sensitivity for trace detection and single-molecule analysis.^5,6^ In addition, spontaneous techniques like Spatially Offset Raman Spectroscopy (SORS),^7^ and the surface-enhanced version (SESORS)^8^ extend the applicability of Raman to subsurface analysis by collecting photons from regions laterally displaced from the illumination point, allowing the investigation of turbid media.^7^ Beyond these, nonlinear Raman techniques such as Coherent Anti-Stokes Raman Scattering (CARS)^9^ and Stimulated Raman Scattering (SRS)^10,11^ enable high-speed, label-free imaging with improved signal-to-noise ratio. Butler et al. summarized in a table the benefits and limitations of the most relevant Raman spectroscopy techniques.^12^

Raman spectroscopy techniques are increasingly applied across diverse fields, including materials science, catalysis,^13–15^ pharmaceutics,^16,17^ biology,^12^ biomedical applications^18^ and diagnostics,^19–21^ forensics,^22^ food safety,^23,24^ surface engineering,^25,26^ sustainability,^27^ security,^28,29^ and cultural heritage,^30^ among others, due to its ability to offer label-free, chemical-specific insights into molecular structures and dynamics. While widely recognized for its versatility and broad applicability, Raman spectroscopy still faces significant challenges that limit its efficiency and widespread adoption. Complex and noisy spectral data, baseline distortions, and overlapping peaks often complicate spectral interpretation, particularly when dealing with heterogeneous multicomponent samples or low signal-to-noise ratios. Reproducibility issues, exacerbated by instrument variability, inadequate environmental/operating conditions, and inconsistent data processing, including calibration protocols and standards, remain key barriers. The increasing volume of data generated with different Raman techniques and approaches in both research and industrial environments further underscores the difficulty and the need of extracting meaningful, actionable insights from large data sets of variable quality. Moreover, the high cost of Raman instrumentation limits accessibility, especially in resource-constrained settings.

The open science movement,^31^ which advocates for transparency, accessibility, and collaboration in research, holds significant potential to address some of these key challenges. Open-source hardware initiatives aim to democratize Raman instrumentation by reducing costs and fostering customizable solutions tailored to specific needs. Similarly, open-source software and computational tools are accelerating innovation in spectra acquisition and analysis by enabling collaborative development of algorithms for particle finding, baseline correction, spectral fitting, and multivariate analysis. The adoption of FAIR (Findable, Accessible, Interoperable, and Reusable) data principles^32^ can play a critical role in overcoming reproducibility issues, as publicly accessible and harmonized Raman databases provide large, high-quality data sets for spectral search as well as model training, validation, and benchmarking. Such platforms not only facilitate data quality, interoperability and reuse as well as transparency, but also promote reproducibility across laboratories, paving the way for robust comparisons and validation of results, increasing trust and applicability. However, finding the path and developing the tools to make data and databases compatible and interoperable is not straightforward. Moreover, even if critical for FAIRness and data usability, data quality is often undervalued, with acceptance criteria not defined or based on arbitrary or subjective parameters.

The integration of the most recent advances in data science and, more specifically, in artificial intelligence (AI), which includes machine learning (ML) as a key component, into Raman spectroscopy complements these open science efforts by offering innovative solutions to the listed challenges. The possibility of collecting large data sets and making them FAIR allows artificial intelligence algorithms to improve, automate and accelerate data acquisition, as well as processing and quality control tasks. On the one hand, AI models might help optimize experimental parameters such as acquisition time, focusing distance, or number and position (see Section 4.4) of measurement points, as well as decide when repetitions are needed, improving both the quality and efficiency of Raman measurements. On the other hand, AI may minimize human errors, identify subtle spectral patterns, detect trace components, and analyze complex multicomponent samples with unprecedented accuracy and speed. Thus, the integration of AI in Raman spectroscopy enables enhanced real-time monitoring of complex biological and chemical systems,^33^ being increasingly used to automate and improve the efficiency of Raman-based analytical tools. The combination of AI techniques with open and FAIR databases enhances reproducibility, accelerates model training, and ensures robust performance across diverse data sets, ultimately bridging the gap between data acquisition and actionable insights, such as informing subsequent decision making, guiding diagnostics, process control, safety assessment, etc. However, large amounts of data are needed, while access to open, ideally FAIR, databases is still limited, restricting the applicability of the technology.

The advances of integrating open science principles and AI into Raman spectroscopy extend across various techniques, from spontaneous Raman to enhanced and nonlinear variants. For example, in CARS the nonlinear optical response leads to the generation of an anti-Stokes signal that can be simply detected with a photomultiplier tube or a spectrometer. However, this technique suffers from spurious signals due to four-wave mixing processes, resulting in a nonresonant background (NRB) that distorts the collected spectra and in a quadratic dependence on sample concentration, thus complicating the extraction of the pure vibrational spectra and the quantification of the analytes.^34^ SRS is technically more challenging than CARS, as it requires modulation transfer techniques to extract the small intensity variation onto the pump or Stokes beam, but it is free from NRB^35^ and its signal scales linearly with sample concentration, thus facilitating quantitative measurements.^11^ Both techniques are increasingly used in histopathology and tissue diagnostics,^9^ benefiting from old pattern recognition and signal processing ML techniques and new AI-based methods that enhance spectral unmixing and improve interpretability. Additionally, open science and FAIR principles play a crucial role in harmonizing and linking techniques in multimodal, hyphenated or correlative approaches that combine Raman with other vibrational spectroscopies, such as infrared,^36^ Brillouin,^37,38^ coherent Raman^39,40^ or photoacoustic imaging,^41^ or different analytical techniques such as scanning-electron microscopy^42^ or quantitative phase microscopy.^43^ In summary, the increasing availability of large, curated data sets will support the development of improved AI-driven tools capable of automatically processing and analyzing Raman spectra across diverse modalities, paving the way for a more integrated, efficient, and accessible Raman spectroscopy ecosystem.

We believe that the convergence of FAIR and open science principles with artificial intelligence represents a paradigm shift for Raman spectroscopy, advancing the field toward automated and scalable workflows in a reproducible way. However, greater collaboration and integration across different communities in the field are essential to realizing the full potential of open science and AI. While notable progress has been made through various open initiatives in the field of Raman spectroscopy, these efforts remain largely disconnected and uncoordinated. This perspective aims to critically review the most relevant initiatives according to the scheme shown in Figure 1, as well as to create awareness of the challenges and opportunities, while serving as a call for cooperation among researchers, data scientists, hardware and software developers, metrologists and end users to foster a more efficient, unified and accessible Raman spectroscopy ecosystem.

**Figure 1:**
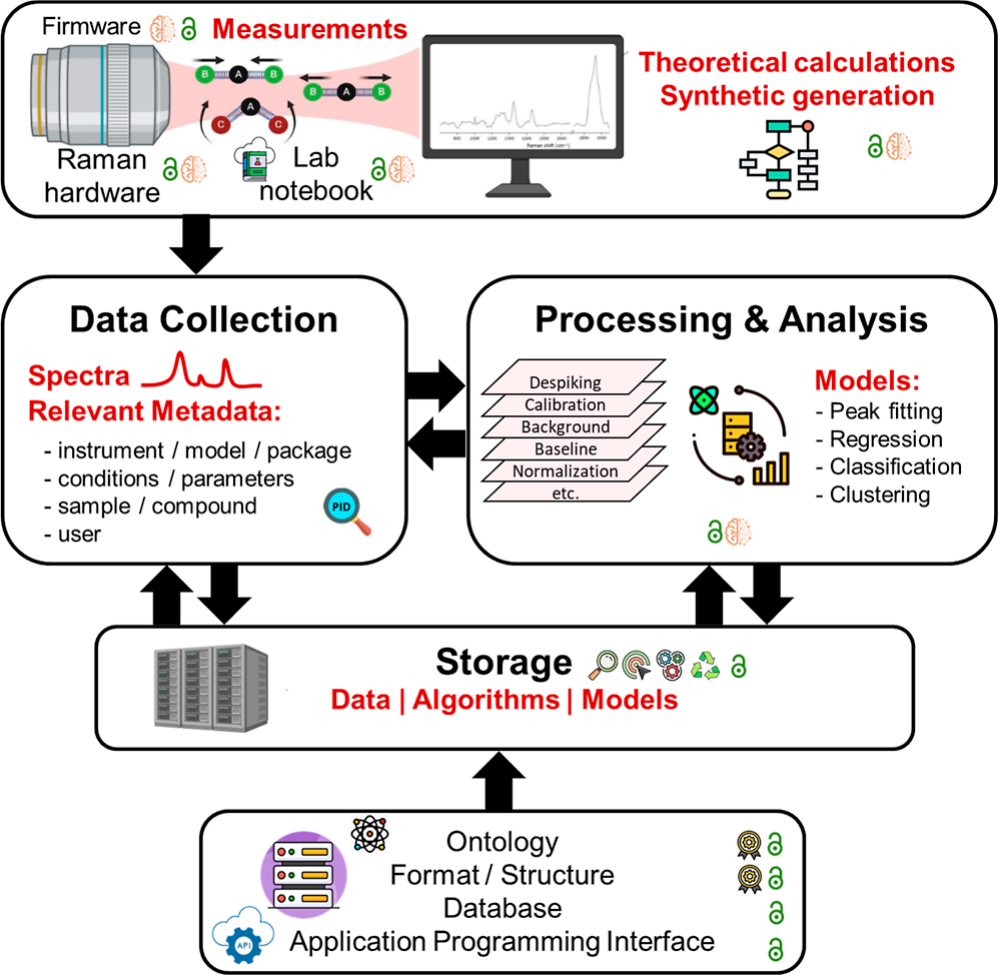
Strategy and key steps toward an open and FAIR Raman spectroscopy environment that embraces artificial intelligence to exploit the full potential of the technique. Raman spectra may be experimentally acquired, simulated through computational models, generated using different artificial intelligence (AI) techniques, retrieved from databases, or even digitally extracted from figures in published literature. Software is used everywhere. Symbols: 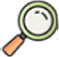 findable, 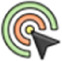 accessible, 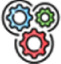 interoperable, 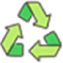 reusable, 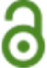 open, 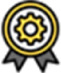 standard, 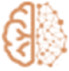 AI, 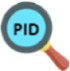 persistent object identifier.

## 2 Open Science and FAIR Principles. Paving the Way toward Digitalization and AI-Driven Data Analysis

### 2.1 Open Science

Scientific research is an inherently iterative process (Figure 2), progressing from initial curiosity to consolidated knowledge, which triggers new questions and hypotheses. This cycle typically begins with (i) observation and hypothesis formulation, followed by (ii) planning and experimental design, and then (iii) the development or application of instrumentation and protocols for sample validation and to ensure methodological rigor for (iv) the performance of experiments, measurements and observations to collect data, ideally according to harmonized, community agreed protocols. This is followed by the (v) analysis of the collected data with the help of software and (vi) reaching conclusions and dissemination of findings through publications. At this stage, the plausibility of the data and whether it substantiates the findings and conclusions is evaluated in the peer review process. However, for modern complex data analysis, often the reviewer will (have to) trust the algorithms and their use. Therefore, these must be cited and accessible, and it is the responsibility of the authors to provide the baseline of quality required in their publications and to establish ways to validate it. The scientific results and evidence are strengthened if data can be replicated and confirmed by several independent researchers. In parallel to the science cycle, the data cycle unfolds. While the research cycle focuses on the advancement of knowledge, the data cycle emphasizes the stewardship of data throughout its lifecycle. Starting with (i) data collection from experiments, calculations, databases, etc. and (ii) analysis, including different pretreatments, processing steps and interpretation, it extends to (iii) data preservation, (iv) harmonization, and (v) sharing, ensuring that data sets remain accessible, interoperable, and (vi) reusable for different purposes. Importantly, data collection and analysis form the intersection between both cycles, reinforcing the idea that high-quality research is inseparable from robust data practices.

**Figure 2:**
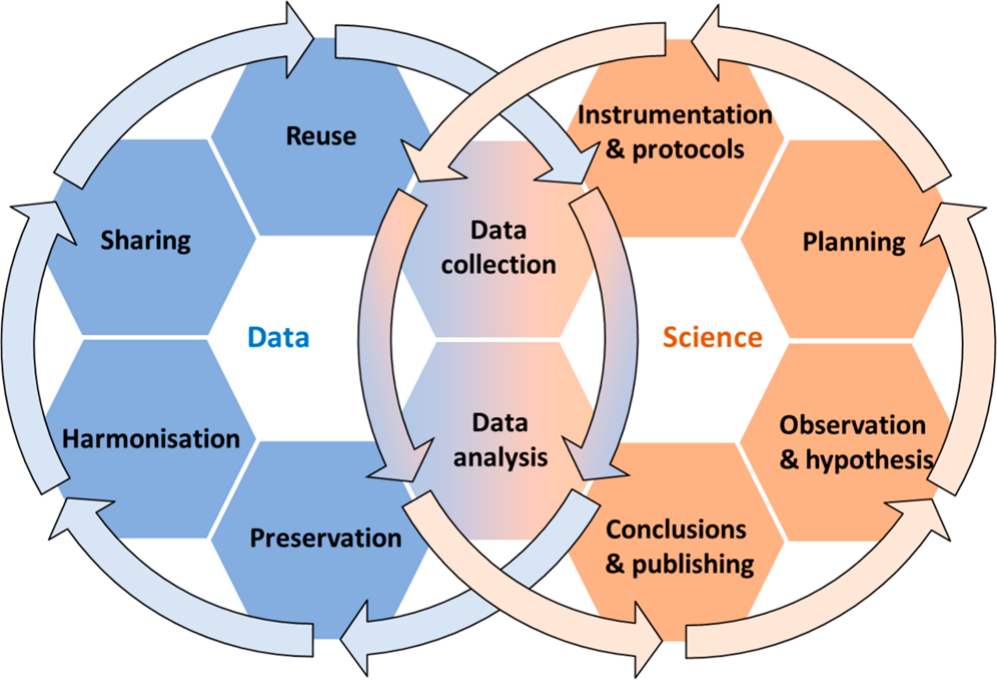
Schematic representation of the research and data cycles, illustrating their parallel and iterative progression and points of intersection. While the research cycle focuses on the generation and communication of scientific knowledge, the data cycle emphasizes the stewardship, preservation, and reuse of data. The shared steps (data collection and data analysis) highlight the critical link between scientific discovery and responsible data management, supporting transparency, reproducibility, and open science.

This close connection between the research and data cycles is central to the implementation of open science. Open science aims to transform research by making it more reproducible, transparent, reusable, collaborative, accountable, and accessible to society. Achieving this level of openness requires that each step of the research process is well documented, made publicly available where possible, and described in a way that allows reuse. When both cycles are aligned with open science principles, they contribute not only to individual project transparency but also to broader goals such as cumulative knowledge-building, interdisciplinary collaboration, and long-term data preservation. In parallel, through the open education movement, open educational resources (OER) are published that contain how to use different analytical techniques, from instrument setup to sample preparation and measurement, including best practices.

Open research and education, thus, ideally involves.

**Open Access (OA)**: Published outputs are freely and unrestrictedly accessible for maximum awareness, use and impact, but the usage is regulated by a license (e.g., CC BY 4.0 ^44^). The first voices in defense of greater openness of scientific knowledge appeared in the mid 20th-century,^45^ but the interest exploded at the beginning of the 90s. For example, arXiv.org, considered the first free scientific archive on the internet, was started in 1991. Currently, open-access publishing is a requisite by most research funding agencies because making research results more accessible contributes to better and more efficient science, and to innovation in the public and private sectors.^46^ The different variants of open access publishing are named using a color code system. Green OA is provided by self-archiving repositories (see the Registry of Open Access Repositories, ROAR),^47^ so it is often based on nonfinal versions and/or requires embargo periods, whereas gold OA is provided by the journal and paid by the authors through the article processing charge (APC). Hybrid journals provide OA if the APC is paid, but also contain closed articles. In contrast, diamond OA is open for both authors and readers.^48^ Open access pertains to the way data or resource is reached or accessed and does not necessarily imply that the accessed data can be used for a particular purpose (see next point “Open Data”). It is possible to have open access with a restricting license that forbids arbitrary types of usage (e.g., data is made available only for education or testing purposes, otherwise the corresponding license must be purchased).
**Open Data**: Documenting and sharing research data openly so they can be used for various purposes. Open data is published under an open license and is often expected to also have an open access, but open data may or may not be granted an open access (see previous point “OA”), as well as it is not necessarily FAIR. Thus, OPENness does not guarantee efficient reuse and is no equivalent to free of charge. “Open” means that once the data is made available (e.g., by free download or upon payment) the owner has freedom to use it according to the granted open license rules.
**Open Notebook**: An emerging practice consisting in documenting and sharing the experimental process of planning, trial and error, analysis, discussion, etc. With the use of electronic laboratory notebooks this can be done in real time, fostering collaboration.^49,50^

**Open-source Software (OSS)**: Documenting research code and routines, and making them freely accessible and available for use, modification and redistribution from its original design. Source code is often handled via public repositories, such as the GitHub platform, that allow developers to create, store, manage and share their code. Similarly to the concept of Open Data, Open-source software must be coupled with a corresponding license^51^ that defines how the developed software can be attributed and used in various levels: redistribution, modification, integration, new code derivation, etc.^52^ Typically, open-source implies obligatory open access to the original source code as well as an open access to all modifications and derivations, however the way the code is used is strictly defined by the license. Also, open-source code does not necessarily mean free of charge software and does not prohibit selling the software (most often the software is sold together with a set of services).
**Open-source Hardware (OSH)**: Documenting designs, materials, and other relevant information related to hardware, and making them freely accessible and available. As in OSS, OSH refers to making the design of licensed physical objects publicly available so that they can be studied, modified, distributed, made, and sold by anyone. The formal definition was written by the open hardware community in 2010,^53^ and is maintained by the nonprofit Open Source Hardware Association (OSHWA).^54^ In 2016, the Global Open Science Hardware^55^ community tailored the OSHWA definition to the specific needs of science, and in 2021, the UNESCO Open Science Recommendation^56^ became the first policy document to include OSH as a component of open science. Open Science Hardware (OscH) includes standard lab equipment as well as auxiliary materials, such as sensors, reagents, analogue and digital electronic components. In comparison with OSS, the OSH source is more complex, as it entails text, binary files and software, also including: schematics, blueprints, logic designs, Computer Aided Design (CAD) files, etc. Best known examples of OSH are Arduino and Raspberry Pi.

### 2.2 FAIR Data

Besides “OPENess”, “FAIRness” has also appeared as a pressing need for research data. The ‘FAIR Guiding Principles for scientific data management and stewardship’ were first published in 2016 ^32^ and how to interpret and implement them has been since then a concern.^57^ The push for FAIR data gained traction led by the GO-FAIR initiative.^58^ FAIR means Findable, Accessible, Interoperable, and Reusable, especially in the context of efficient machine usage, machine actionability and automation, and thus not only for humans but also “For Artificial Intelligence Ready”^59^ The four basic FAIR principles are further fine tuned with a set of sub-principles^60^ revolving around key concepts such as: rich metadata, ontology annotations, persistent identifiers, machine actionability (e.g. an API access to the data), data licencing, efficient and adequate data models, etc.

The target for FAIR data is: (i) well documented data: the metadata must stick to the data, if possible from its creation and all through its life cycle, and ideally within the same file (or data resource bundle). For this reason, the use of hierarchical file formats is highly encouraged (see Section 6). (ii) Standardized data (and metadata): to ensure interoperability, it is essential for the scientific community (of a given field and/or a given technique) to agree on a data format, including the representation, file format and unit of the data and metadata, the minimal set of metadata required or recommended to contextualize the data, the minimal set of data required or recommended to be retained over time as well as the duration of this retention. (iii) Data that live in a place where they can be called/explored/compared across projects/laboratories/institutes. To that aim, the archiving platform must enable filtering and searching by using the standardized metadata fields. It must be known by the community, or findable otherwise, for example by being integrated in a known network of intersearchable repositories, or referenced in a known data catalogue (containing the essential metadata of its entries, but not the full entries). Ideally this platform needs to include tools for data visualization, comparison, exportation (see Section 2) and validation. (iv) Data that can be not only compared with results from the same technique, but also combined with results from other techniques. This requires that not only the tool-specific metadata follows standards defined by the technique expert (e.g., Raman spectroscopy), but that the field-specific (medicine, biology, chemistry, material science, etc.) metadata also follows field-specific standards. Moreover, having metadata based on a strong ontology allows to connect more easily across fields (see Section 6.2).

FAIRness, on the one hand, allows access to open science for artificial intelligence/machine learning, and on the other hand, is a time-saving strategy for confidential information located and retrieved inside a company^61^ and prevents data starvation. However, there are some general obstacles and barriers that obstruct data FAIRification: inertia from the past, bad data management practices, lack of understanding of FAIR principles, lack of incentives, too high price or huge resources needed, ineffective collaboration between data generators and data managers. Moreover, although the FAIR principles provide a generic frame and guidance for data management, the FAIRification process requires domain-specific solutions. While the FAIRification transition takes place, huge amounts of non-FAIR scientific data are generated. The main sources of unFAIRness are.

- F: Metadata inconsistency, missing metadata, lack of standardized metadata and persistent identifiers.
- A: complex licensing and authentication processes, lack of reliable and scalable storage infrastructure.
- I: lack of standardized data structures, ontologies and vocabularies, data spread across various repositories without proper linking mechanisms, lack of efficient tools for automation and API access.
- R: ambiguous or restrictive licensing terms, lack of detailed documentation/metadata.

Valuable tools for the FAIRification process are the laboratory information management systems (LIMS) and electronic lab notebooks (ELN),^62^ software that helps to document experiments, and that often has features such as protocol templates, collaboration tools, support for electronic signatures and the ability to manage the lab inventory. Their use may boost transparency and reproducibility by capturing workflows digitally, facilitating data sharing and collaboration. ELN software can be open-source, like eLabFTW,^63,64^ and even the notebook itself can be made open (see “Open Notebook” in previous section). ELNs integrate well with AI systems,^65^ enabling automated analysis and fostering a smarter, more efficient approach to data analysis and interpretation. However, their adoption in academia, although increasing, is not yet as widespread as predicted^66^ due to difficulties such as adapting well established workflows, the wide variety of ELNs^67^ and the cost and time needed for their implementation without a direct and clear reward. Therefore, despite the opportunity offered by ELNs, most data sets are still based on simple SQL or similar enhanced datasheets.

## 3 Open and FAIR Practice Implementation

The experience of open and FAIR practice differs across academia, business, and instrument companies.

### 3.1 Academia

In academia, many funding organisations state as a requirement of funding that data generated during a project must be published ‘as openly as possible, but as closed as necessary’. However, not all analytical science areas have dedicated data repositories, making it difficult for others to find these data, and most instruments save files in proprietary formats, with inconsistent export options, often missing valuable metadata. The lack of standards, particularly in novel techniques and modalities, hinders metadata harmonization. Another barrier to uptake is that many data analysis packages are only able to read specific proprietary formats, and more generic packages only able to read files in open formats. Finally, academic work is being put under a growing ‘publish or perish’ pressure, creating very strong incentives to fast publication over proper data documentation.^68^ While the long-term benefit of FAIR data is clear, researchers often lack the chance to consider long-term goals. Furthermore, it is unfortunate but true that many researchers and research institutions do not have the support of a data manager and lack the knowledge to produce FAIR data, which is perceived as a complex task as well as a burden. As a result, it is crucial for FAIR data to be implemented in an automated manner.

### 3.2 Private Sector

In the business arena, FAIR is often confused with open, and therefore dismissed as requiring the release of commercially sensitive information, particularly metadata that may contain lots of detail. In addition, companies may have no budget for this, but existing in-house practices that their workforce are familiar with, and are reluctant to change. However, the adoption of FAIR principles, even in a closed environment, can lead to better records retention and make it easier to share data in-house, between different laboratories, and across sites. Also, if companies are acquired or merged, FAIR practice enables the data systems of each partner to be combined more easily. Incoming staff are more likely to be familiar with systems and data recording methods, and third-party software, particularly open source, can be easily utilized.

For instrument/software companies the business perspective is somewhat different as there may be a concern that FAIR principles may affect their intellectual property rights, e.g. metadata could allow competitors to determine the operating parameters of an instrument, reducing a commercial advantage. The Raman spectroscopy market is a very sensitive ecosystem which has not received generous funding from the institutions, as the biological field (see Section 3.3), but which has formed itself under fierce competition. Moreover, FAIR and open are frequently misinterpreted with free, and going in the opposite direction as business. For example, the sale of databases is often linked to specific instruments and configurations as well as to specific application fields on purpose to increase benefits; The launch in 2004 of PubChem, which has become a foundational resource in chemistry and biomedical research (see Section 6.4) led to controversy when the American Chemical Society (ACS) lobbied against it, arguing it would undermine their proprietary CAS database. Another example is the data format-software dependency to avoid compatibility with external applications/instruments from competitors. A paradigmatic case is that of spc file format for storing spectroscopic data, invented by Galactic Industries. After the company was purchased by Thermo Fisher Scientific, the format changed and no open protocols for data conversion are available. In summary, business development and implementation of new tools commercially is driven by customer requirements and demand, because it is expensive. Companies are often innovation drivers and make innovation accessible to nonspecialists, but there must be a balance between the risks of open access to FAIR data and tools, and the benefits of better integration and reaching a broader community.

### 3.3 An Example in the Biological Field

All scientific disciplines hinge on the availability of reference data and the tools to efficiently process, share, search, compare, and interpret them. The impact of open science principles implementation by the wide community has been a breakthrough in some fields of research. The successful implementation of open and FAIR principles in molecular sciences, for example, may serve as inspiration for Raman spectroscopy and other scientific fields.

Molecular scientists started to optimize their data sharing models in the early 80s, simultaneously with the advent of the internet. Open molecular databases, such as GenBank^69,70^ and many others^71^ have been linked to a period of remarkable progress colloquially called the “Molecular Revolution”. Arguably, the emergence of GenBank and similar databases has initiated a series of changes that fundamentally transformed molecular sciences, allowing unprecedented progress based on optimized data-sharing strategies. The positive effects on the field of molecular sciences fall into several groups: (i) Effective reuse of data reduces duplicated research, saving finances and time in R&D. An example may be comparative genomics, where the availability of genomic sequences from multiple organisms in GenBank has allowed identification of evolutionary relationships, conserved genes, and regulatory elements, e.g. in noncoding regions in the human genome.^72^ Similarly, effective data sharing in GenBank and GISAID boosted progress in pathogen genomics, aiding fast development of diagnostics, vaccines, and therapeutics, e.g., during COVID19.^73^ (ii) Standardization of data formats (e.g., FASTA) and annotation practices improved data interoperability and reproducibility, which has been critical for large-scale meta-analyses combining data from many sources, e.g., genome-wide association studies (GWAS).^74^ (iii) Development of freely available data-processing tools, largely dependent on standardized data. Tools like BLAST (Basic Local Alignment Search Tool), with adjustable search parameters, can efficiently sort and compare GenBank sequences.^75^ (iv) Collaborative and interdisciplinary research was facilitated by a centralized repository accessible to scientists of different specializations. Human Genome Project (HUGO) represents a good example, as it enabled global collaboration thanks to the data shared via GenBank.^76^ Another such area is cancer genomics, since The Cancer Genome Atlas (TCGA) data in GenBank enabled researchers worldwide to study cancer mutations and identify potential therapeutic targets.^77^ (v) Data-driven research was enabled by the efficient work with large data sets, shifting the paradigm from hypothesis-driven research. This opened the area of systems biology, leading to a ground breaking work on the gene networks, using transcriptomic data from GenBank to map key regulatory pathways.^78^ (vi) Machine learning models for analysis of large omics data sets have been developed into highly efficient tools for prediction of gene functions, protein interactions, and disease outcomes.^79^ Effective use of AI in combination with large molecular databases such as GenBank resulted in a successful protein folding prediction model, yielding the Nobel Prize for chemistry in 2024.^80,81^ (vii) Ethical and policy questions have been raised thanks to the open-access nature of GenBank, such as data ownership and privacy, with positive impact on scientific practices. Data sharing policies were updated—journals and funding agencies now often require researchers to deposit genomic data in public repositories like GenBank as a condition of publication or grant approval.^82^ Ethical considerations involving the sharing of human genomic data has led to the development of guidelines for responsible data sharing.^83^ (viii) Dissemination, education, and training has been influenced, as bioinformatics courses at universities incorporate GenBank data into their curricula.^84^ Citizen science platforms like Foldit and Zooniverse use publicly available data to engage the public in scientific research, democratizing access to omics data.^85^ The effects of this transformation are summarized in Table S1.

## 4 Open Raman Instrumentation: Hardware and Control Software

Raman spectroscopy systems have emerged as pivotal analytical instruments with a frenetic market evolution. Butler et al. made a list of commercial instruments available by 2016 and the corresponding operational software.^12^ Since then, many new instruments have been launched to the market. The private industry and hospital market for Raman technologies, including nonlinear approaches, is already a reality: the NIO system from Invenio Imaging has received CE-mark and FDA Breakthrough Device Designation; Cambridge Raman Imaging develops coherent Raman microscopes and coordinates the EU CHARM project to translate them into clinical practice; and RiverD instruments are widely applied in industrial dermatology. However, on the one hand, there is a need for more affordable and accessible Raman instruments,^86^ as the relatively high costs associated with commercially available Raman instruments may present a barrier to their accessibility for many academic institutions and companies, hindering a broader usage. On the other hand, several groups developing Raman scattering related techniques use home-built systems composed of different commercial and/or homemade parts that typically allow for higher performance, tunability and versatility than commercial systems. This is particularly relevant for the most recent Raman-based techniques, such as nonlinear and coherent Raman imaging approaches, which still require a high degree of customizability and whose optimal configurations are not yet well established within the research community. While this is a very common practice (in contraposition to regular users who typically utilize commercial instruments), in general, researchers do not publish instructions or documentation related to their setups (both hardware and control software) more than a few paragraphs in some PhD thesis and scientific papers (usually not intended to be reproduced). Furthermore, customization does not have a clear audience and researchers might target the creation of a future start-up company, protecting and keeping the intellectual property (IP) for potential development. In fact, some companies hold relevant patents for Raman-based setups that block their open access, for example related to SORS^87^ (patented by STFC, now controlled by Agilent), sequentially shifted excitation (SSE) for fluorescence removal (by Bruker^88,89^ ), or tuneable lenses for autofocus (by SERSTECH).^90^

Open-access research and education resources can help laboratories close knowledge transfer gaps ensuring that lab-relevant resources stick around even after a subject matter expert has left and also the gap between basic and highly technical guides. This might have a positive effect in general data quality, as setup alignment^91^ and measurement protocols directly impact quality parameters as SNR (see Section 6.5). In addition, FAIR data may contribute to the knowledge, as the metadata might describe the instrument quite extensively, but not enough to compromise industrial secrets. In fact, what metadata is mandatory to ensure trustable results is a very critical question. Open Raman hardware and control software aim to fill these knowledge gaps. Given these challenges, it is crucial for the research community to work toward a convergence on the most effective solutions, particularly in the field of coherent Raman techniques. Establishing clear protocols and guidelines for the development of these setups in laboratory environments would greatly enhance reproducibility, facilitate adoption, and accelerate advancements in the field.

### 4.1 Advanced Home-Built Raman Systems

Several studies have explored methodologies for constructing Raman spectroscopy setups, particularly for spontaneous Raman applications. For instance, Palonpon et al. provide a comprehensive guide to setting up Raman and SERS microscopy for molecular imaging of live cells.^92^ Paudel et al. examine customized Raman configurations for pharmaceutical applications, focusing on improving measurement accuracy.^17^ Smith & Dent offer an in-depth analysis of Raman spectroscopy hardware configurations, including modifications for academic use.^93^ More recently, Domingos et al. propose a low-cost Raman spectroscopy setup integrated with machine learning to enhance spectral analysis.^94^ Regarding back focal plane (BFP) imaging,^95^ a Fourier technique providing data in reciprocal space used to investigate Raman radiation patterns^96^ and how they are modified by plasmonic particles,^97,98^ Vasista et al. presented the possible configurations utilized to project the back focal plane of the objective lens onto an imaging camera or to a spectroscope, also discussing the typical sources of error and possible ways to overcome it.^99^ While these studies provide valuable insights into spontaneous Raman setups, there remains a significant gap in standardized methodologies and open-source documentation for coherent Raman techniques, highlighting the need for further research and collaboration in this area. A few papers have been published describing detailed instructions on how to modify a commercial microscope^100^ or to build a nonlinear microscope from scratch^101^ for coherent Raman microscopy; however, there is not just a single experimental setup/modality accepted in the literature as the best for neither SRS nor CARS, so that the user should also decide which technique to implement and find the best strategy for implementing it by reading relevant literature, which often do not contain all the details for replicating the setup.

### 4.2 Low-Cost Solutions

There are also some Web sites showing instructions on how to build basic systems based on second hand components^102^ and publications on do-it-yourself (DIY) spectrometers for education,^103–105^ but only a few initiatives provide more detailed information to build the actual systems such as CAD files, schematics, tutorials, etc.^106–109^ Currently, these are mostly unipersonal projects (no platform for team collaboration is provided) and applications seem to be still limited. However, with easily available cheap lasers, sensitive cameras, and the possibility of 3D printing components such as Raman probes^86^ low-cost Raman spectroscopy gradually gains interest in academia, industry and the DIY community. This is apparent from the growing number of web pages, video tutorials, and articles dedicated to building homemade Raman spectroscopes, as reflected in Table 1.

**Table 1:** Instructions, Know-How, and Inspiration for Building Various Low-Cost and Compact Raman Spectrometers for Education, Research, Diagnostics, Miniaturization, Automation, etc. Published in the Literature or Online Sources^a^ ^b^

Medium (link)	year	Description/main components	Material costs (USD)	Resolution (cm^–1^)	Geometry	Excitation λ (nm)	Laser power (mW)	Ref
Article	1992	N_2_, MC(1), PMT (scan)	<12,000	26	90°	337	2	^110^
Article	1994	Port.: HeNe, MC(2), APD (scan)	NA	8	0°	632.8	9	^111^
Article	1995	HeNe, MC(2), PMT (scan)	11,000	27	90°	632.8	10	^112^
Article	1998	Ar^+^, EF, C-T MC, PMT (scan)	NA	20	180°	514.5	500	^113^
Article	2002	DPSS, NF, com. SPM + CCD	<5000	70	90°	532	10	^114^
Article	2004	SERS: LP, NF, com. OF SPM + CCD	3668	10	180°	532	4	^115^
Article	2008	DPSS, NF, com. SPM + CCD	6500	NA	45°	532	20	^116^
Article	2010	SERS: LP, NF, com. OF SPM + CCD	5000	20	180°	532	4	^117^
Article	2010	LP/Ar^+^/HeNe, NF, com. SPM + CCD	4000	NA	90°/180°	532/515/633	5/30	^118^
Article	2015	LP, LF, EF, OF, rDG, DSLRc	3000	15	0°	532	<100	^119^
Article	2017	SHRS: DPSS, EF, CBS, 2× rDG, PC	NA	38–55	90°	532	<300	^120^
Article	2018	3D, DPSS, LF, EF, C-T, l-CCD	836	NA	180°	532	150	^105^
Article	2020	DL, OPU, EF, OF, com. SPM + CCD	730–1100	NA	180°	520	50	^121^
Article	2021	DPSS, EF (optional), 4× L, tDG, PC	50+phone	50	45°/90°	532	50	^122^
Article	2021	LP, color. Glass EF, com. SPM + CCD	690	30	180°	532	70	^103^
Article	2021	DPSS/Ar^+^, EF, tDG, DSLRc	NA	NA	90°	532/488	100	^123^
Article	2022	DPSS, EF, com. SPM + CCD	>4650	NA	90°	532	5	^124^
Article	2024	DPSS, NF, OF, com. SPM + CCD	3074	7.5	180°	532	200	^86^
Review	2022	miniature Raman instrument for SERS-based PoC testing						^125^
Web	2023	Open Raman: Step-by-step guide to DIY low-cost Raman spectrometer						
Web	2021	RamanPi: Open source 3D printable Raman spectrometer w. RaspberryPi						
Blog	2019	DIY Raman spectrometer blog w. notes on Toshiba TCD1304 linear CCD module						
Blog	2025	DIY Raman spectrometer tutorial and notes (from the author of Open Raman)						
Video	2017	Video: DIY Raman spectrometer						

a The outlined Raman systems are described in terms of their main components, price, spectral resolution, laser excitation wavelength and power, and system geometry.

b Abbreviations: 3D: Based on 3-D printed parts. APD: Avalanche photodiode. Ar^+^: Argon-ion laser. CBS: Cube beam-splitter. Com. (OF) SPM + CCD: Commercial (optical fiber-coupled) spectrometer with CCD detector. C-T: Czerny-Turner (monochromator type). DL: Diode laser. DPSS: Diode-pumped solid-state laser. DSLRc: Digital single-lens reflex camera. EF: Edge (long-pass) filter. HeNe: Helium–Neon laser. l-CCD: Linear CCD (e.g., TCD1304). L: Optical (condenser or collimating) lens. LF: Laser-line filter. LP: Laser pointer. MC; MC(1); MC(2): Monochromator (single; double). N_2_: Nitrogen laser. NA: Not available. NF: Notch filter. OF: Optical fiber. OPU: Optical pickup unit (from CD or DVD player). PC: (Smart)­Phone camera. PMT: Photomultiplier. Port.: Portable instrument. rDG; tDG: Diffraction grating (reflective; transmissive). SERS: Surface-enhanced Raman spectroscopy. SHRS: Spatial heterodyne Raman spectroscopy.

### 4.3 Raman-Enabled Cell Phones

A promising, yet challenging^126^ approach relies on mobile phone cameras as spectrometers.^127,128^ Smartphone-based spectrometers are designed to meet several critical criteria, as pointed out in Figure 3a, while aiming to be modeled after a simple spectroscope (see Figure 3b,c as example).^129^ Cell phones continuously develop to enhance our experience of interaction with our surroundings. The growing number of communication channels, databases, and AI-based algorithms provide us with increasingly efficient means to move around, satisfy our needs, and make important decisions. Lately, phone manufacturers started to include more niche functionalities, such as thermovision (e.g., UleFone Power Armor 18T Ultra), Geiger counter (e.g., SoftBank Pantone 5 107SH), laser range finder (e.g., Doogee S97 PRO), LiDAR (e.g., iPhone 12 Pro), and even a near-IR spectrometer allowing rudimentary chemical analysis (Changhong H2). At the same time, there is a demand for miniature hand-held Raman instruments,^125^ which is reflected by the emergence of companies such as BaySpec, Lightnovo and more. These current trends could soon merge and allow integration of Raman spectroscopic modules into cell phones. Lightnovo has filed a patent^130^ describing, among other designs, an external Raman-spectroscopic module for a cell phone: a thin, wedge-shaped box, attached to the rear side, using the phone camera as the spectroscopic detector. Some experimental Raman designs are especially well-suited for miniaturization and integration into self-contained units with all dimensions in the order of millimeters.^120^ This leaves virtually no more technical barriers to the advancement of Raman-enabled cell phones. Such devices would provide the user with a powerful tool to make more informed decisions and to perform curiosity-driven exploration.

**Figure 3:**
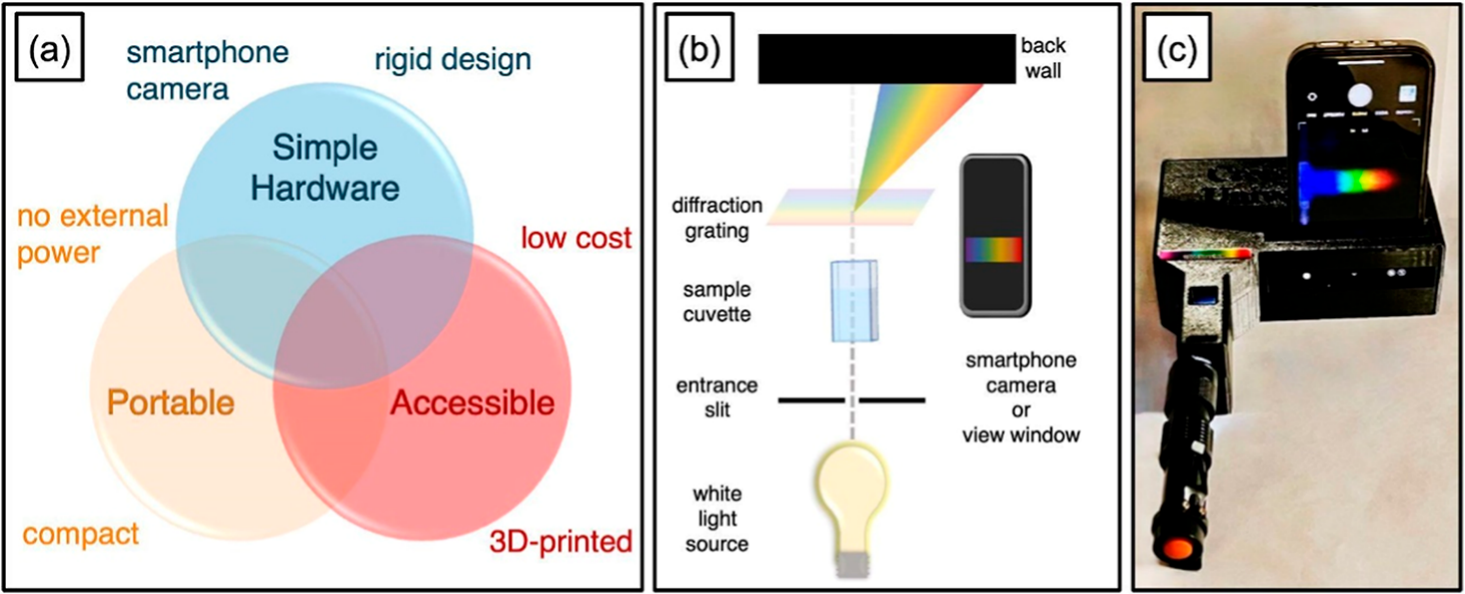
Three-dimensional (3D)-printed spectrometer design planning. (a) Governing design principles. (b) Schematic of the spectroscope-inspired, polychromatic spectrophotometer design. (c) A photograph of CU Spec used as a spectroscope with a smartphone (iPhone 13) installed for viewing. Reprinted with permission under a Creative Commons CC BY NC License from Nguyen, T.; Tobin, N.; Bournef, H.; Gross, E. M.; Destino, J. F. A Portable, Spectroscope-Inspired Three-Dimensionally Printed Smartphone Spectrophotometer. 2025, 3 (1), 1–8. https://doi.org/10.1177/27551857251314525. Copyright 2025 The authors. Published by Sage journals.

While keeping data quality in terms of signal-to-noise ratio and spectral resolution remains a challenge associated with miniaturization, the combination of smartphone-based spectrometers^120,122^ with cheap and easy-to-use SERS^125,131^ substrates is specially envisioned for portable point-of-care testing.^132^ SERS smartphone-based devices hold the promise for enabling highly sensitive measurements of trace chemicals such as pesticides in agricultural products, pollutants in water, or preliminary medical self-diagnostics of various metabolic disorders, infections, or even cancer. These analyses would mostly hinge on automated matching of the user’s spectra to dedicated spectral libraries. Following open and FAIR principles will therefore play a large role in such developments.

### 4.4 Instrument Control

Commercial Raman instruments are typically accompanied by proprietary control software, such as WiRe (Renishaw), LabSpec (Horiba), or Control (Witec), which often include the option to purchase additional paid, typically expensive, modules for advanced features. Home-built Raman setups can rely on custom-built software developed in platforms such as LabVIEW, Matlab, or Python. However, such custom software is rarely shared with the scientific community, not necessarily due to a lack of willingness to share but rather because these tools are tailored to highly specific hardware configurations and not well documented, limiting their applicability to other systems. Regarding the open hardware instruments described in the previous section, only a few come along with open control software. For example, the Spectrum Analyzer Suite was designed to acquire, save, filter and calibrate spectra taken on the OpenRAMAN spectrometer, but will also work on any dispersive spectrometer based on PointGrey cameras, and extensions for other camera manufacturers can be developed using the provided DLL files.^133^ An interesting solution is NOMAD CAMELS,^134^ a standalone, open-source software that controls experiments and acquires FAIR-compliant data from different measurement instruments directly at the origin and with rich metadata.^135^ Measurement protocols can be configured without programming knowledge and can easily be adapted to new research questions at any time. Micro-Manager is a free and open source microscope control software^136,137^ that in combination with Pycromanager^138,139^ represents a nice alternative for Raman microscope control. Halfway we can find Spectragryph,^140,141^ a free (for academic, private and noncommercial use), but not open, general-purpose, optical spectroscopy package for the acquisition, plotting, processing and analysis of data from spectrometers.

The operation of any software-controlled Raman spectrometer can be automated to work autonomously and optimize data acquisition for balanced spectral quality and use of resources. In this regard, López Reyes and Rull Pérez developed a suite of algorithms for the automation of the Raman instrument onboard the ExoMars rover that may be adapted to any spectrometer. The algorithms adjust the acquisition parameters to the sample characteristics dynamically for the proper management of detector saturation, fluorescence, and data quality.^142^ In a different approach, the quest for microplastics detection has also triggered the development of software for finding particles on surfaces such as filters based on images, to automatically select the points of the filter to be characterized by Raman (and other) spectroscopy.^143,144^ An alternative to expensive commercial particle recognition software tools, open tools have also been implemented combining particle detection (after acquisition of optical images), spectroscopic directed measurements, and even spectral search and data analysis, as for example Gepard Enabled PARticle Detection (GEPARD)^145^ or TUM-Particle Typer.^146,147^ While these softwares (both open and commercial) work relatively well for particles on rather clean and homogeneous backgrounds, there is hope that AI based tools might help to properly identify particles in more complicated and diverse backgrounds and improve identification accuracy in the case of weathered plastics. AI-based tools could also be developed to manage Raman instrument acquisition more efficiently. Very recent advances include, for instance, SlitNET, a deep learning enabled spectrometer slit which allows to reconstruct Raman spectra with enhanced resolution from low-resolution inputs,^148^ or DeepeR, deep-learning enabled high-throughput Raman imaging through the combination of fast denoising and reconstruction of low signal Raman signatures via deep learning with fast spatial super-resolution via neural network.^149^ Also, recent advances in AI-assisted programming suggest that tools such as GitHub Copilot or Mahari AI could be leveraged to speed up the development of custom Raman control software, facilitating integration across heterogeneous hardware and improving usability. Although these tools may not immediately generate fully functional systems, they can support rapid prototyping, code documentation, and modularity. Similar AI-assisted approaches have already been explored in other domains, such as laboratory automation,^150^ industrial process control,^151^ and robotics integration.^152^

## 5 Open Software and AI for Raman Spectral Data Extraction, Processing and Analysis

Software is or can be used in every step of Raman spectroscopy characterization, from experiment design,^153^ sample size planning^154^ and data acquisition to spectra (pre)­processing, information extraction and results interpretation. Artificial intelligence is anticipated to revolutionize Raman spectroscopy by optimizing and automating all these steps.

### 5.1 Raman Data Collection

Raman spectra may be obtained from different sources and by different procedures that will be more or less adequate depending on the final use: (i) experimentally acquired (see Section 4, operating instructions and best practises), (ii) simulated through computational models^155–158^ (check in the curated list of open databases^159^ those containing simulated spectra, there are also open-source packages for calculation of Raman spectra, as AiiDA, based on density-functional theory and the electric-enthalpy functional^160,161^ ), (iii) generated using different mathematical approaches,^162^ including artificial intelligence (AI) techniques (see Section 5.3), (iv) retrieved from databases (see Section 6.4), or even (v) digitally extracted from figures in published literature. Images of Raman spectra in the scientific literature are the most frequently available form of reference data. However, their use as such has obvious limitations. Even basic visual comparison with data from other sources is frequently complicated, e.g. by different orientation and range of the *x*-axis, image size, and aspect ratio. While open science may enhance data accessibility in current and future publications, accessing data from older sources remains a challenge. One viable solution is the extraction of spectral data from plot images using suitable software. Data quality (relative to the original) depends on the parameters of the image and the extraction process. There are at least two free and efficient tools for data extraction from images of Raman spectra: Web Plot Digitizer^163–165^ is an online service, accessible after free registration, offering a suite of tools for extracting data from images of various types of graphs, including line graphs such as Raman spectra. ImageJ^166,167^ is an open-source, freely available software for image analysis, offering a range of functions that allow extracting data from images of Raman spectra. The basic workflow involves the following steps: Open the image, navigate to Image–Adjust–Color Threshold to isolate the spectral curve of interest, and then select Analyze–Tools–Analyze Line Graph to extract and save the data for further processing. ImageJ2 and Fiji, both based on ImageJ, are also suitable for this purpose. These tools provide researchers with accessible and efficient methods to convert spectral images into useable reference data when the original data are unavailable.

### 5.2 Raman Data Preprocessing and Analysis

Chemometrics is the science of extracting information from chemical systems (typically represented with analytical data) by data-driven means^168^ using tools ranging from classical statistical techniques, formal logic and multivariate methods^169^ to state-of-the-art artificial intelligence,^19,170,171^ including machine learning^153,172–174^ algorithms, which are increasingly pivotal for unravelling complex data sets (see Section 5.3). Raman spectroscopy often requires advanced preprocessing and modeling to extract meaningful insights, particularly in techniques like SERS, with strong variations in intensity and spectral profiles caused by different sample-SERS substrate orientations,^174^ as well as in complex applications like biological systems^175^ or thin films, where the Raman signal is superimposed by those of the substrate and the different layers,^176^ and optically anisotropic materials.^177^ Especially in the latter one, the Raman signal depends not only on the polarization of the excitation and detection but rather on the orientation of crystal or investigated material. A typical Raman spectra preprocessing pipeline may follow this sequence:^178^ spike removal, intensity and wavenumber calibration, noise reduction/smoothing,^179^ baseline subtraction, and normalization. These steps are aimed at obtaining a clean, high-quality spectral data set, facilitating modeling and enabling the identification of key components and spectral differences.^180,181^ However, the optimal order and number of steps may be discussed, and excessive or inappropriate preprocessing can distort spectral features and introduce artifacts, potentially leading to misleading conclusions. For instance, Wahl et al. recently showed that the selection of preprocessing techniques can influence the spectral representation of Raman features in brain tumor tissue.^182^ This highlights the need for careful consideration when comparing spectral data across different Raman studies, as well as the importance of methodological transparency and detailed reporting of preprocessing procedures. An interesting tool is ASpecD,^183^ a modular framework written in Python for recipe-driven analysis of spectroscopic data without programming skills that focuses on reproducibility and good scientific practice (Figure 4).

**Figure 4:**
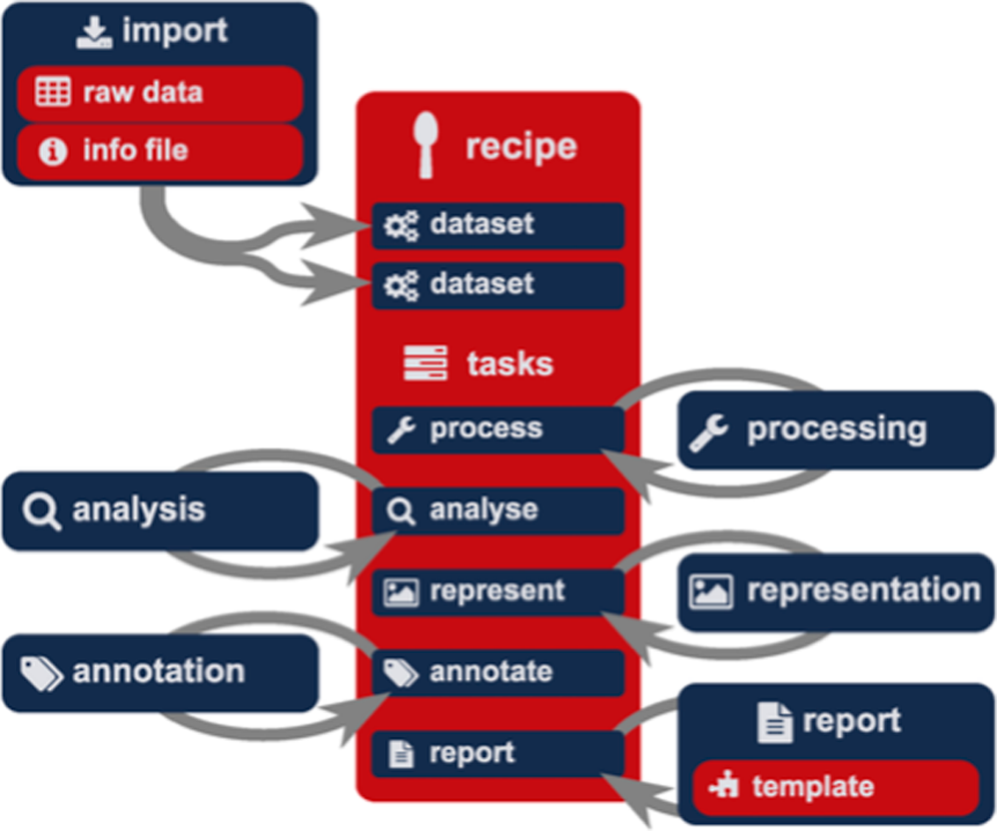
Recipe-driven data analysis as implemented within the ASpecD framework. Recipes provide a level of abstraction that empower users not familiar with programming to describe the details of arbitrarily complex data analysis step-by-step in a formalized way. They can be thought of as a special kind of user interface. A recipe consists of a list of data sets to operate on and a list of tasks that shall be performed. Tasks can be everything implemented within a package based on the ASpecD framework, from simple processing steps to complex reports. Reprinted with permission under a Creative Commons CC BY NC ND License from Popp, J.; Biskup, T. ASpecD: A Modular Framework for the Analysis of Spectroscopic Data Focusing on Reproducibility and Good Scientific Practice**. Chem. Methods 2022, 2 (6), e202100097. https://chemistry-europe.onlinelibrary.wiley.com/doi/10.1002/cmtd.202100097. Copyright 2022 The Authors. Published by Wiley-VCH GmbH. All rights reserved, including rights for text and data mining and training of artificial intelligence technologies or similar technologies.

The increased availability of computing power and of many application programming interfaces (APIs) available in commercial multivariate data analysis software, but particularly of AI open source libraries such as Scikit-learn,^184^ TensorFlow,^185^ Keras^186^ or PyTorch^187^ have triggered an intense advancement of chemometrics in recent years, which significantly accelerated progress in Raman data science. A curated collection of over 90 open-source packages and tools related to Raman spectroscopy is available at GitHub,^188^ demonstrating the breadth of the community’s contributions. Among these there is open-source code for specific preprocessing steps like for example peak fitting,^189^ spike removal^190^ or baseline subtraction,^191^ as well as libraries containing most preprocessing steps and analytical tools. Recent examples of open-source python packages are ramanchada2,^162,192^ RamanSPy,^193^ PyFasma,^194,195^ Open Raman Processing Library (ORPL)^196^ and PyRamanGUI.^197^ Ramanchada2 poses a particular emphasis on calibration and harmonization of spectra and spike removal algorithms comparison,^198^ as well as synthetic Raman spectra generation, being able to load files in many different open and proprietary formats including.spc (see Section 3.2 for information on this format), sp, spa, wdf, ngs, jdx, dx, txt, csv, and rruff. Figure 5 displays some of its functionalities. RamanSPy has been developed to provide a comprehensive suite of tools for Raman spectroscopy analysis, integrating preprocessing, visualization, and AI-based data modeling. Besides preprocessing tools, PyFasma offers two multivariate techniques (PCA, PLS-DA) and spectral deconvolution within a modular framework. ORPL implements a baseline removal algorithm validated from multiple data sets acquired in human tissue and biofluids and provides a graphical user interface (GUI). PyRamanGUI combines common analysis methods with the organization of Raman data in projects and the plotting of spectra through a graphical user interface written in Python. Orange Data Mining^199^ is an open-source software designed for data visualization and machine learning. While not specifically developed for Raman spectroscopy, it offers a modular framework that allows for spectral data analysis, chemometric modeling, and integration with machine learning workflows, making it an adaptable tool for Raman data scientists. There are also interfaces for the handling of spectra written in programming languages other than Python, as for example Fityk^200^ for peak fitting purposes, written in C++, or hyperSpec,^201^ written in R, which combines tools for chemometric data analysis of hyperspectral data plus the integration with further information such as spatial information, time, concentrations, etc. Open Specy^202,203^ focuses on microplastic analysis, it is available on web or as an R package and provides an extensive spectra library combined with ML classifiers. In addition to these open-source tools, several nonopen-source but freely available platforms provide accessible solutions for Raman data processing and analysis. RamApp,^204^ for example, is a web-based platform enabling the simple and coding-free analysis of hyperspectral data (Raman images and Raman maps), offering essential preprocessing functions such as baseline subtraction and peak detection, along with multivariate and spectral matching tools to facilitate data interpretation and data visualization. HYPER-tools^205^ is a software based on MATLAB for chemometrics with a focus on hyperspectral data.^206^ Another notable project is RAMANMETRIX,^207^ a platform designed for Raman spectral data analysis with a focus on automation and usability. All these tools, whether fully open-source or just open-access, demonstrate the growing role of computational resources in democratizing Raman spectroscopy and enhancing its analytical capabilities.

**Figure 5:**
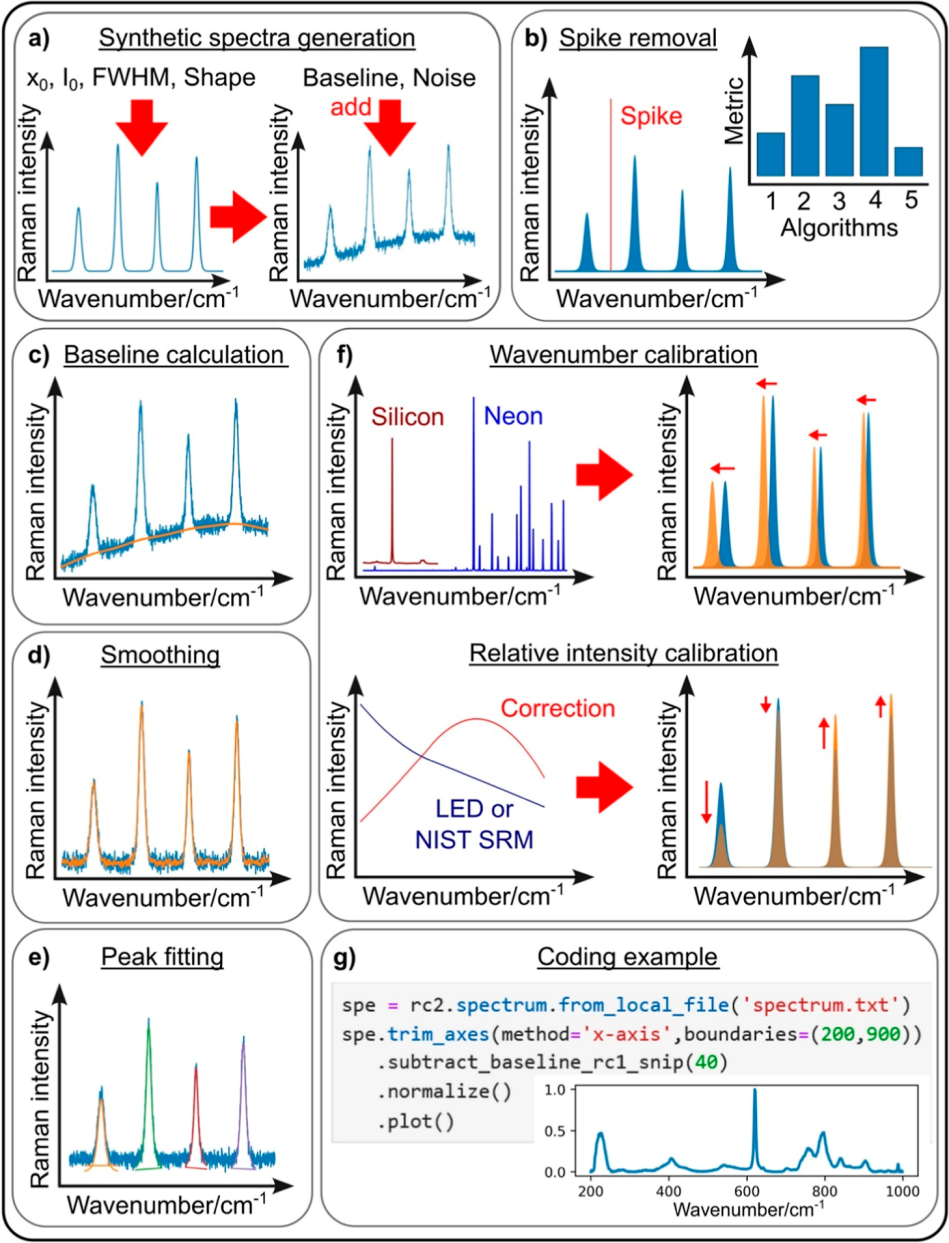
Some functionalities of ramanchada2, an open-source Python library for Raman spectra processing. (a) Synthetic data generation, (b) spike removal algorithms and comparison tool, (c) baseline subtraction, (d) spectra smoothing, (e) peak fitting, (f) instrument calibration, and (g) code example. Reprinted with permission under a Creative Commons CC BY License from G. Georgiev, N. Coca-Lopez, D. Lellinger, L. Iliev, E. Marinov, S.Tsoneva, N.Kochev, M. A. Bañares, R. Portela, N. J. Open-Source for Raman Spectroscopy Data Harmonization. J. Raman Spectrosc. 2025, 56 (9), 878–881. https://doi.org/10.1002/jrs.6789. Copyright 2025 The Authors. Journal of Raman Spectroscopy published by John Wiley & Sons Ltd.

The growing number of initiatives not only reflects the vitality of the field but also its fragmentation. Many tools are developed independently rather than extending existing ones, sometimes due to the lack of awareness, other times because prior code might be difficult to understand, poorly documented, or not designed with modularity in mind. In most cases, researchers may find it easier to implement new functionalities from scratch than to adapt or contribute to existing libraries. While this approach can foster innovation, it also risks redundancy and hinders interoperability across tools, highlighting the need for more collaborative development practices and community agreed methodologies.

### 5.3 Artificial Intelligence in Raman Spectroscopy

Artificial intelligence can improve data collection (see Section 4.4), preprocessing, modeling, and interpretation. In the context of SERS, AI also holds promise for optimizing substrate design, selecting suitable SERS configurations, and engineering tailored Raman reporters for specific applications.^170^ Moreover, AI might also be used for semantic data ingestion and data set harmonization, as Hapich et al. demonstrated with data sets related to microplastics and trash.^208^ AI can also be FAIR; a concise and measurable set of FAIR principles for AI models has been proposed in the literature that could be adapted to the field of vibrational spectroscopy (see Section 6.3) as the author did for accelerated high-energy diffraction microscopy.^209^ Figure 6 shows the publication trend in Raman spectroscopy, both in general and explicitly combined with the use of algorithms and/or artificial intelligence (including in AI the concepts of machine learning, deep learning, and neural network). While the total number of Raman spectroscopy publications has steadily increased over the decades, those mentioning AI have surged in recent years, especially after 2015. The inset highlights this trend, emphasizing the rapidly growing role of AI in the field.

**Figure 6:**
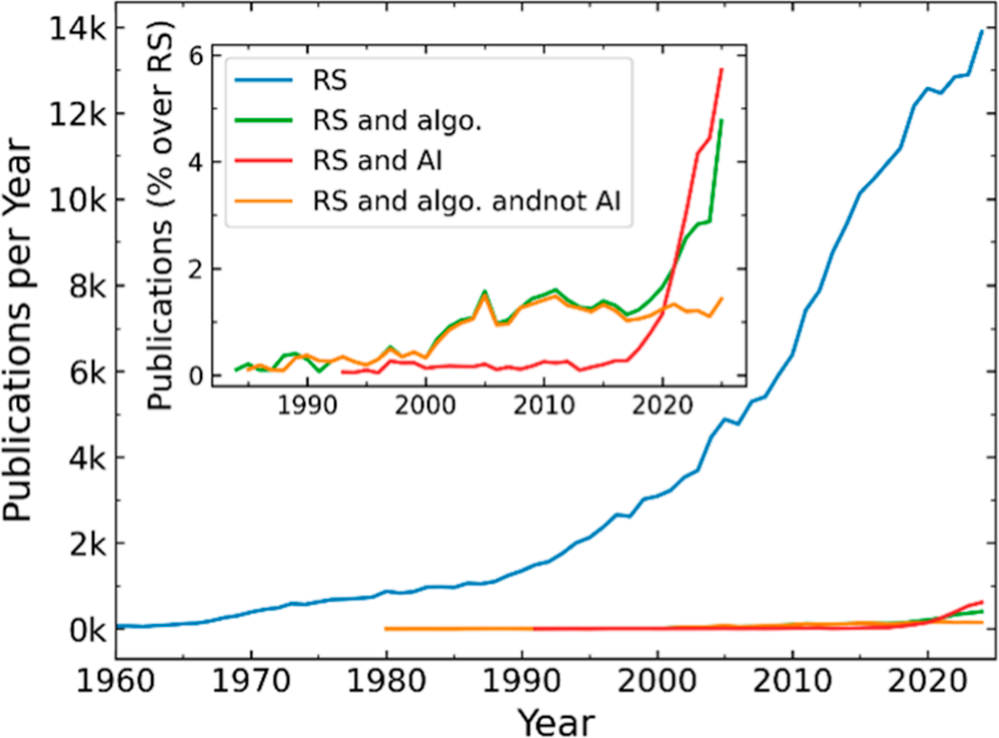
Annual number of publications (1960–2024) containing selected keywords in the title, abstract, or keywords. The blue line shows publications including the term “Raman spectr*” (RS). The green line represents publications mentioning both RS and “algorithm” (algo.). The red line corresponds to publications containing RS together with at least one artificial intelligence-related term (AI) among “artificial intelligence”, “machine learning”, “deep learning”, and “neural network”. The orange line shows the subset of RS and algorithm excluding artificial intelligence-related terms. Inset: same data represented as the percentage of total publications per year related to Raman spectroscopy.

While classical machine learning methods such as Partial Least Squares (PLS), Support Vector Machines (SVMs), and Random Forests remain relevant and useful, particularly when used with engineered spectral features,^33^ modern implementations of AI are increasingly used. AI applications in Raman spectroscopy initially focused on classification, regression, peak identification, and segmentation and/or automating the preprocessing steps. Convolutional Neural Networks (CNNs) are used for spectral classification and feature extraction.^210^ Other deep learning architectures—Recurrent Neural Networks (RNNs) and ResNets—were introduced for sequential spectral data modeling, while Generative Adversarial Networks (GANs) showed early potential in data augmentation and denoising.^210,211^ Recent advances in foundation models, such as Deep Spectral Component Filtering (DSCF), demonstrate how self-supervised pretraining can enable generalizable solutions across diverse spectral analysis tasks, including denoising, quantification, and interpretation, even in complex biological matrices.^212^ Lately, generative AI is also emerging as a promising approach in Raman data analysis. Flanagan and Glavin^213^ compared various data synthesis techniques, demonstrating how GANs and Variational Autoencoders (VAEs)^214^ enhance Raman classification accuracy by generating realistic synthetic spectra. Guo et al.^215^ provide a comprehensive review of AI-driven approaches in spectroscopy, coining the term Spectroscopy Machine Learning (SpectraML) to describe this emerging field, and propose a roadmap for the future of SpectraML, emphasizing the integration of multimodal data, the development of foundation models for spectroscopy, and the application of AI in real-world chemical and biomedical diagnostics. The paper categorizes SpectraML methodologies into two primary tasks: forward modeling (molecule-to-spectrum prediction) and inverse modeling (spectrum-to-molecule inference). In the forward problem, AI models predict spectral data from molecular structures. Traditional methods rely on vector-based molecular descriptors, while more advanced approaches leverage Graph Neural Networks (GNNs) and physics-aware models to enhance prediction accuracy. A recent example is Mol2Raman by Sorrentino et al.,^216^ which uses a Graph Isomorphism Networks with edge features (GINE)-based GNN to predict Raman spectra directly from SMILES (Simplified Molecular Input Line Entry System) representations, achieving high accuracy across diverse molecular structures and enabling real-time predictions via a web interface (see Figure 7). This model, trained on over 31,000 DFT-calculated spectra, outperforms previous approaches and demonstrates the scalability and interpretability of GNN-based spectral prediction. In the inverse problem, deep learning architectures, including transformers and sequence-to-sequence models, are applied to reconstruct molecular structures from spectral data. The increasing role of VAEs and GANs in both tasks, enabling synthetic spectrum generation and improving model robustness, is remarkable. In a different generative-AI approach, large language model (LLM)-based conversational assistants such as ChatGPT can help the assignment of measured spectra to molecules or molecular mixtures and navigating this complex ecosystem following the example of the BioImage.IO Chatbot, a community-driven platform leveraging GPT-4, retrieval augmented generation (RAG) and advanced reasoning frameworks such as ReAct to help users navigate bioimaging tools, databases and services and eventually solve bioimage analysis tasks.^217^

**Figure 7:**
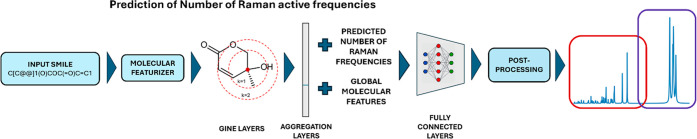
Representation of the Mol2Raman architecture schema for predicting Raman activities for every Raman shift. This architecture also employs the predicted number of Raman-active frequencies as well as the global molecular descriptions provided by the Daylight and Morgan fingerprints. The red and blue boxes in the spectrum represent the predictions in the fingerprint (500–2100 cm^–1^) and C–H region (1900–3500 cm^–1^) with an overlapping region of 200 cm^–1^. Reprinted in part with permission under a Creative Commons BY NC ND 4.0 License from Sorrentino, S.; Gussoni, A.; Calcagno, F.; Pasotti, G.; Avagliano, D.; Rivalta, I.; Garavelli, M.; Polli, D. Mol2Raman: A Graph Neural Network Model for Predicting Spectra from SMILES Representations. ChemRxiv 2025. https://doi.org/10.26434/CHEMRXIV-2025-67395. Copyright 2025 The authors. Published by ChemRxiv.

Despite all the advances, several challenges have constrained the effectiveness and applicability of AI in Raman spectroscopy.

**Data Availability**: The reliance on small, proprietary data sets limits model robustness, and transparency remains a concern.^218^ The lack of large, open, Raman data sets is a significant limitation for pretraining and transfer learning opportunities.^218^ An interesting option to overcome this issue is data augmentation through the generation of synthetic data with tools such as ramanchada2,^162,192^ which implements a rich toolset for synthetic spectra generation with user-defined peak positions, intensities, and shapes (any profile available at lmfit library 3, as well as any user-defined function or arbitrary array).
**Data Quality and Preprocessing**: Raman spectra are often noisy and require extensive preprocessing, including baseline correction and denoising. Typical preprocessing steps require setting a threshold or the adjustment of one or more parameters, depending on the employed algorithm. For example, spike removal algorithms might require a width^190^ or a z-scores^219^ threshold, baseline subtraction might require one parameter when using SNIP^220^ or two when using ASLS,^221^ etc. Novel approaches are proposed to, for example, estimate spectral baselines,^222^ smoothing, or even handling all steps simultaneously.^223,224^ Deep learning models trained directly on raw data have shown promise in bypassing traditional preprocessing steps, improving end-to-end learning efficiency.^225^ Noise, considered detrimental in classic machine learning, is now recognized as beneficial in deep learning. Techniques like dropout inject noise to regularize models and reduce overfitting,^226,227^ while denoising autoencoders train models to extract robust features from corrupted inputs.^228^ Theoretical work has shown that noise can improve learning dynamics by shaping the optimization landscape and enhancing generalization,^229^ see for example the recent review by Song et al.^230^ The discovery of grokking—where models first overfit and then suddenly generalize-further emphasizes the complex role of noise and training dynamics^231^ (for more details see also Section 6.5).
**Interpretability and Transparency**: The “black-box” nature of deep learning models raises concerns about trust and interpretability.^232–235^ Explainable AI (XAI),^236,237^ techniques such as saliency maps, and SHAP (Shapley Additive Explanations) are being integrated to enhance model interpretability, particularly in biomedical applications^238,239^ and slowly gaining attention in the spectroscopy domain.^240^ To improve AI performance in Raman spectroscopy, researchers have begun incorporating physical constraints into deep learning models. These physics-informed neural networks integrate domain knowledge, reducing the need for large data sets and improving model generalization. In Simulation-Assisted Learning, for instance, Gastegger et al.^241^ introduced a model that leverages external field interactions to enhance Raman spectral predictions. Similarly, Chen et al.^242^ proposed an approach using ab initio molecular dynamics data to train deep learning models, accelerating Raman spectral simulations with improved accuracy. In the case of Hybrid Models, the studies combine physics-based approaches with AI-driven predictions. For example, Zhang et al.^243^ integrated AI with SERS for bacterial identification in blood samples, achieving high classification accuracy even in complex biological environments.
**Generalization and Domain Shift**: Deep learning models trained on specific data sets often fail to generalize due to differences in instrumentation, sample preparation, and environmental conditions.^218^ This might be due to overfitting and/or domain shift (also called “dataset shift”),^244^ where variations in experimental setups lead to performance degradation when models are applied to new data.^213^ Blake et al.^218^ discuss training/test data set split at the level of spectra vs split at the level of subject/sample, which is a classic case of multi-instance learning (MIL).^245^ Data leakage^246^ challenges are well recognized in the machine learning community with various solutions developed during the past decade.
**Data Representativity**: Training AI or ML algorithms requires gathering data sets which—while being very large—contain representative and comparable inputs. This brings two challenges: (i) having enough and consistent information (metadata) on our data for comparability, (ii) choosing between strictly filtering less relevant data out from the training data set, with the risk of reducing its size, or including the metadata together with the data for the algorithm to decide, with the risk of overparametrizing the problem. An intermediate approach is to keep the metadata out but weight the training data as a function of its quality and relevance.^247^ The representativity question brings us to the question of the publication bias: negative results for a given application are much less likely to be published than positive ones.^248–250^ Applied to machine learning, this means that the set of data available for AI training is typically not representative of reality, leading to overoptimistic conclusions by the trained AI (see Figure 8). Data set shift techniques are available to help in such situations.

**Figure 8:**
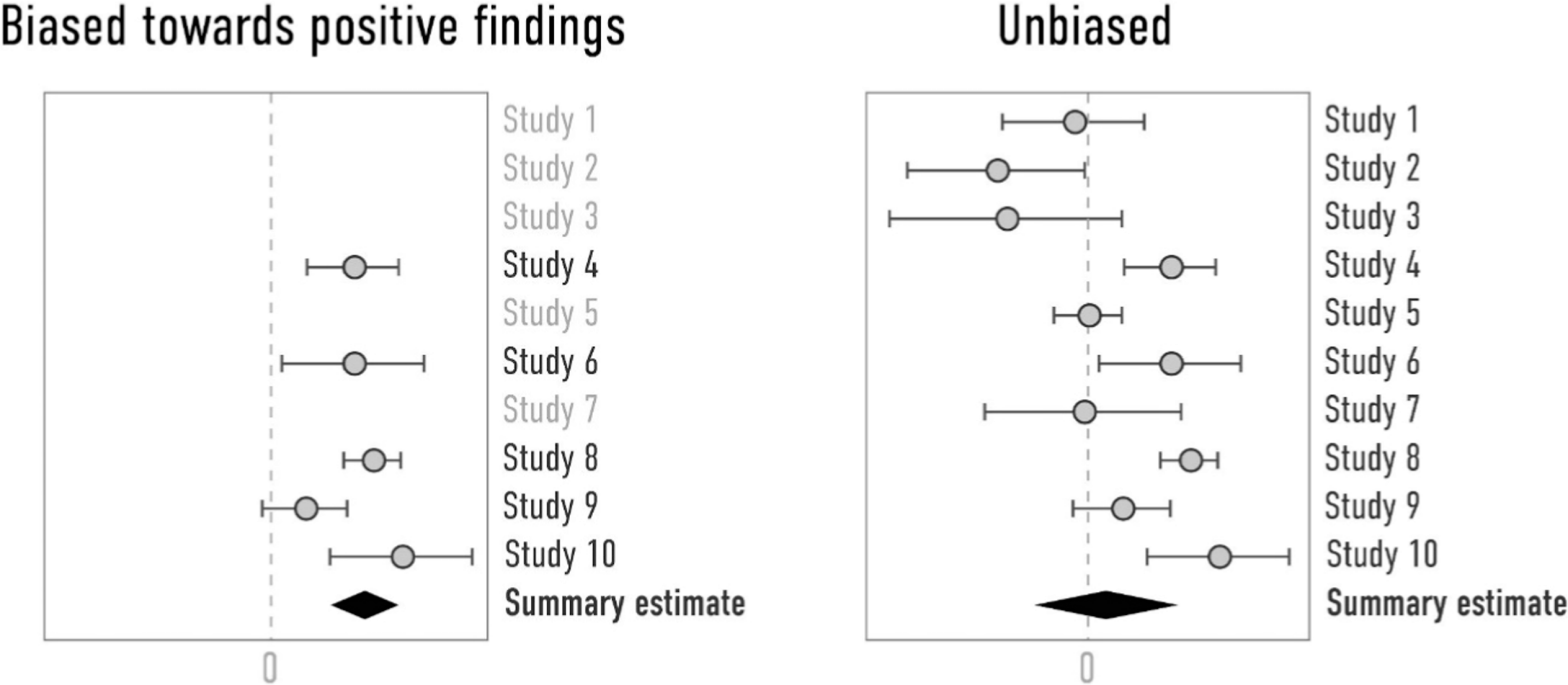
Schematic illustration of the publication bias and its effect on meta-studies conclusions from https://www.researchequals.com/modules/w0d6-9kwa, copyright CC BY 4.0 Meike Latz. The same effect is expected to happen in machine learning.

In their review paper, Guo et al. discussed the key challenges in SpectraML, particularly the lack of large, high-quality, and diverse spectroscopic data sets, which limits generalizability, highlighting the need for standardized benchmarking data sets and exploring emerging solutions such as self-supervised learning and physics-informed AI models to address domain shifts.^215^ Besides data augmentation with artificially generated and simulated spectra, the application of open and FAIR principles to existing data (see Section 6) is crucial to face the above-mentioned problematics: while openness increases the available amount of data for training algorithms, FAIRness offers enough metadata for proper contextualization—and ideally in a standardized format—thus giving us the elements to judge the comparability of data sets, and filter accordingly (or include the metadata as inputs to the algorithm). Moreover, those concepts are associated with a strong paradigm change in which data are valuable per se, and can be published on their own, and given unique identifiers that enable to acknowledge the authors for their work, even when the application results do justify writing an article. As a result, open and FAIR data have a very strong potential to reduce the effect of publication bias.

Integrating AI models into established chemometric workflows and regulatory environments poses additional hurdles, especially in fields like pharmaceuticals where regulatory standards are stringent.^251^ However, even in these fields the power of AI models is blooming in the past decade, e.g. in drug design and pre-screening stages. Finally, there are ongoing debates about reproducibility, as generative AI will not produce twice the same results, and about FAIRness of AI-driven methods, emphasizing the need for transparency, standardized protocols, and open-access tools to ensure equitable and reproducible applications. Addressing these challenges is critical for realizing the full potential of intelligent methodologies in Raman spectroscopy.

## 6 Open and FAIR Raman Data Storage. Standardisation, Quantity, and Quality

The increasing volume of Raman spectroscopy data has made standardized data management and sharing crucial for scientific progress. Contemporary scientific endeavors are achieved by multidisciplinary efforts performed by a variety of research groups and institutions; hence, the variety of generated data sets is the natural outcome. The paradigm of big data makes it impractical that each group manages the “full” database—the latter would demand extreme data redundancy and problems with data update and version synchronization. The efficient way to handle big data is by means of distributed and federated systems where the data resources are spread across multiple locations/network nodes and only a portion of the metadata is duplicated for interoperability and linking purposes. The full might of FAIR principles is a must for efficient, transparent federation of heterogeneous data sets, as well as smooth work across them. Federated systems require efficient, comprehensive, standardized and flexible data models at the core of the data management software platforms, which emphasizes the importance of defining a semantic data model.^252^ Reproducibility challenges in Raman spectroscopy are seasoned with a diverse array of proprietary data formats (see examples in, e.g.,^162^,^192^ ) and inconsistent metadata descriptions across different instruments and laboratories, leading to significant barriers in data integration and reuse. The FAIR principles provide a framework for improving research data management (see Section 2), but their implementation in spectroscopy requires careful consideration of domain-specific needs. Successful data sharing in Raman spectroscopy depends on the ability to capture not only the spectral data itself but also the complete experimental context, including instrument configurations, measurement conditions, and data processing workflows, including calibration. Just note that the use of AI does not require a precise calibration of the instrument when models are to be built with and applied to data from the same instrument collected during a stable time interval. However, a precise calibration will minimize possible errors such as making decisions based on subtle changes caused by modifications in the state of the machine or measurement conditions as well as allow using data from several machines and model transferability. Without standardized ways to represent and share this information, reproducing experimental results or combining data sets from different sources becomes impractical. See a FAIRification checklist for Raman spectroscopy data in Figure 9.

**Figure 9:**
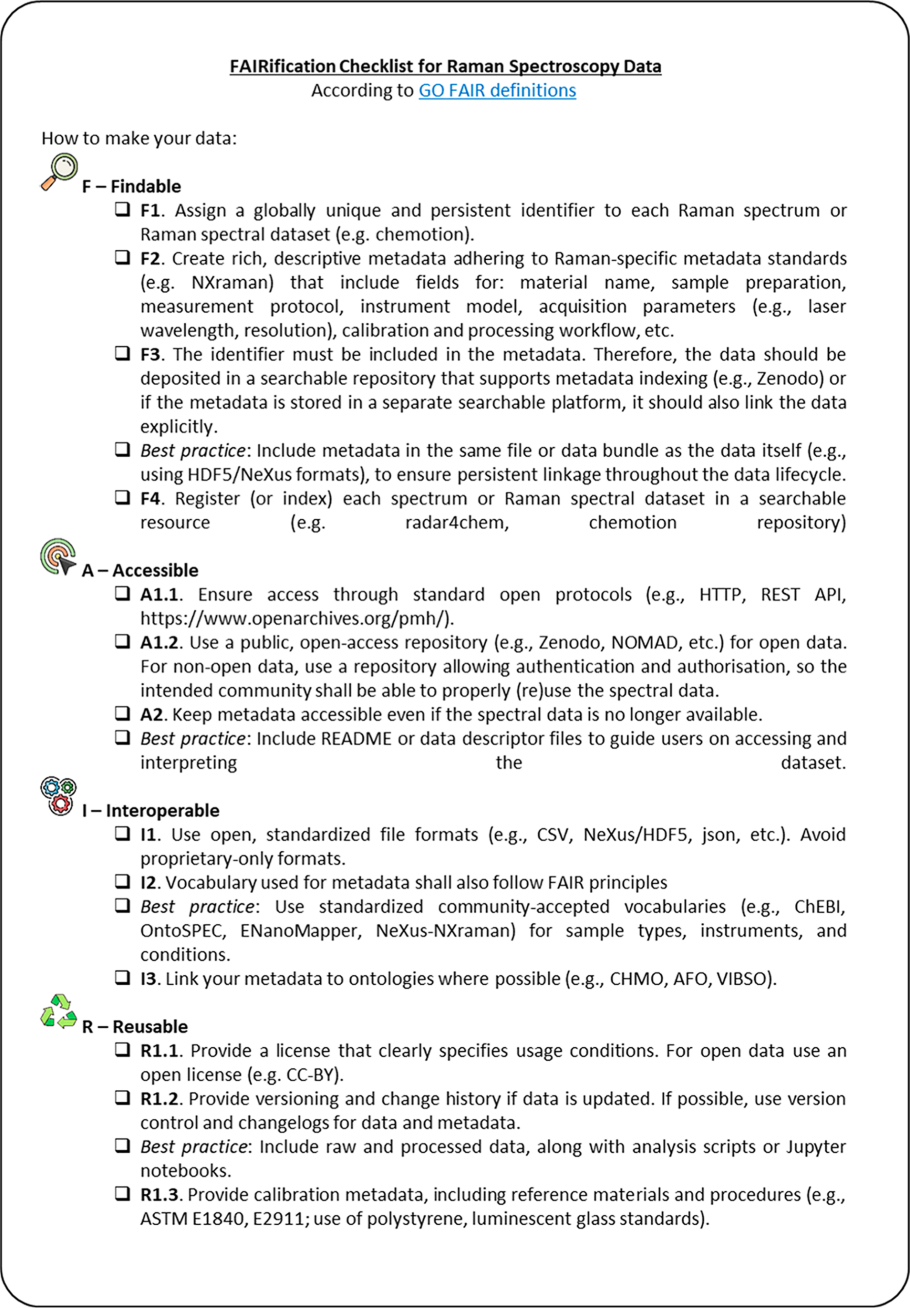
Checklist for Raman spectroscopy data.

Hence, a fundamental requirement for FAIR reproducible Raman spectroscopy is data harmonization using standardized procedures (see Section 6.1) and the standardization of FAIR data formats and metadata descriptions (see Sections 6.2 and 6.3). Generic metadata de facto standards such as schema.org’s Data set vocabulary and the DCAT Application Profile (DCAT-AP) provide established frameworks for data set discovery and cataloguing^174,253,254^ and, thus, enable broad interoperability with general-purpose search and indexing systems, thereby increasing the visibility of research data. However, their generic nature means they lack the granularity needed to describe crucial Raman experimental details. For example, while these standards can indicate that a data set contains Raman spectra, they cannot effectively expose specific measurement parameters like laser wavelength or power settings that are essential for systematic searches and reproducibility. The development of FAIR databases (see Section 6.4) for Raman spectroscopy, therefore, requires a multilayered approach to standardization. At the foundation, domain-specific semantic standards must provide precise terminology for describing experimental parameters and procedures. These semantic standards then inform the development of standardized data formats that can capture both raw data and its experimental context. Finally, databases and data sharing platforms must implement these standards consistently to enable reliable data integration and reuse. However, to fully exploit the potential of Raman spectroscopy in a variety of applications and the power of artificial intelligence in the field, an additional critical point is ensuring the fulfilment of data quality and quantity requirements (see Section 6.5).

### 6.1 Raman Data Calibration and Harmonization

Raman spectroscopic data intrinsically depend on how the Raman signal is obtained (instrument and acquisition configuration as well as sample, sample preparation and measurement environment) and (pre)­treated/processed to generate and analyze the Raman spectra. However, there is consensus neither within academia nor among standardization bodies or manufacturers on the best way to calibrate Raman instruments and harmonize Raman data. The calibration frequency and protocol depend on the type of Raman instrument, target application/user, or availability of references, for example. Moreover, the need for the user to calibrate, verify and maintain the Raman instrument can be a daily challenge and a source of deviations. Standards, in particular those related to instrument calibration and validation, should serve as a reference for instrument manufacturers, industrial users, and researchers, providing guidelines to ensure data robustness. Standardization in Raman spectroscopy has become increasingly important as the technique expands in both academic and industrial applications to ensure data consistency and reproducibility. However, as indicated in the comprehensive review of the existing standards, guides and practices for Raman spectroscopy by Ntziouni et al.,^251^ the standardization landscape remains fragmented, with protocol gaps and outdated guidelines as well as a limited range and availability of certified reference materials for calibration. We have compiled a curated list of existing and under development standards related to Raman spectroscopy, including documents and certified reference materials, classified by topic.^255^ It shows that the standardization efforts in Raman spectroscopy have been largely led by the American Society for Testing and Materials (ASTM) and national metrology institutes, such as the National Institute of Standards and Technology (NIST) of the United States or the National Metrology Institutes of Japan (NMIJ) and China (NIM), but also a number of institutes and companies offer certified materials for this purpose.^256^ Besides, the pharmaceutical industry, one of the largest industrial users of Raman spectroscopy, provides through the most predominant pharmacopoeias Raman-specific recommendations for calibration, validation, and measurement procedures, often referencing ASTM documents and NIST-certified reference materials.

Calibration operations relative to the *x*-axis are covered in several standard protocols and methods, which are not interrelated and do not always coincide (Figure 10).^251^ These operations include wavelength/wavenumber assignment to the detector pixels and Raman shift calculation defined by the laser line (ASTM E1840); spectral dispersion over the observation window that drives the valid spectral range and, in turn, the pixel resolution; spectral resolution (ASTM E2529); and other pixel adjustments. Y-axis calibration is less frequent and worse understood, but relative intensity correction is essential to compare spectra acquired with different instruments or excitation wavelengths. Only a limited number of manufacturers offer relative intensity correction procedures using external or internal references, typically based on ASTM E2911. These references can be wavelength-specific luminescent glasses certified by metrology institutes or a calibrated radiation source, like tungsten lamps or the innovative radiance light emitting diode (LED) sources introduced by ELODIZ in 2024.^256^

**Figure 10:**
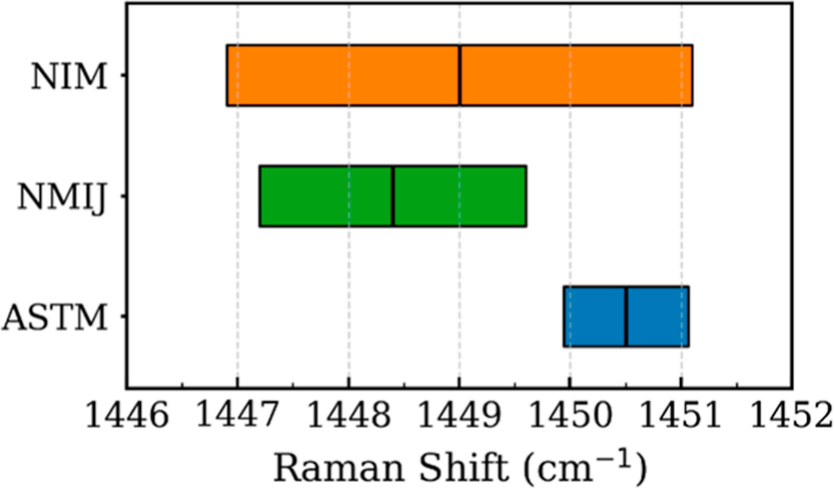
Variation of the position and tolerance values given for the 1450 cm^–1^ asymmetric peak of polystyrene in the NIM GBW13664 and NMIJ RM8158a certificates and in ASTM E1840.

The effect of the optical path, reference material, operator, or algorithms on the calibration accuracy is unclear, as well as that of factors such as the use of polarisers, sampling geometry, focal point volume, penetration depth, dispersion, detector-related factors (integration time, saturation, gain, etc.), fluorescence or luminescence effects, electronic noise, environmental light contamination, cosmic radiation, etc. Areas that require particularly close attention are, for example, low-resolution portable analysers, techniques like SERS, or quantification. In this line, absolute chemical concentration can be determined using SRS, where signals scale linearly with molecular concentration.^257^ This requires collecting not only the signal at specific Raman shifts but a continuous SRS spectrum over a large spectral region, ideally the entire vibrational spectrum, followed by spectral unmixing^258^ for proper calibration and background removal. Current quantitative methods rely on calibration curves of SRS intensity versus concentration, assuming identical measurement conditions for samples and standards. However, determining absolute chemical concentration remains challenging due to: (i) light scattering, which reduces both the amount of light reaching the focus and the collected signal; (ii) non-Raman background signals from transient absorption, photothermal effects and cross-phase modulation, which must be minimized or corrected.^259^ To solve these problems, internal standards for light scattering correction can be used, employing ratiometric measurements to compare SRS signals from different vibrational peaks to cancel out scattering and absorption effects, such as the “Normalized Raman Intensity” (NoRI) approach^260^ for biological applications. Interestingly, the absolute SRS cross section (σ_SRS_),^261^ measured in Göppert-Mayer (GM) units, the same as two-photon absorption (TPA) cross sections, has been introduced, allowing for a direct comparison of SRS with the spontaneous Raman counterpart. This framework reveals that SRS responses can match or even exceed those of TPA for certain molecules, providing deeper insights into molecular properties and their role in scattering processes. This advancement will enable more precise and effective quantitative SRS imaging.

The international community, including researchers, manufacturers, and regulatory bodies, must work together to modernize the standardization landscape, introducing new protocols and updating outdated guidelines. Long-term initiatives such as the Versailles Project on Advanced Materials and Standards (VAMAS) provide the technical basis for harmonization of measurement methods, leading to best practices and standards, and the technical working area (TWA) 42 is actively devoted to Raman spectroscopy with several projects.^262^ They have identified the major obstacle in uptake of Raman techniques as the lack of traceable quantification, primarily due to absence of standards. Recently, the CHARISMA project focused on improving Raman data harmonization and, among other activities, proposed a comprehensive protocol, currently being tested in framework of VAMAS, for: (i) wavenumber and intensity calibration considering the full optical path, (ii) resolution assessment across the entire spectral range, (iii) calibrated instruments’ twinning by using a new reference material to harmonize absolute intensity values.^263–265^ Micro- and nanoplastics analysis, a hot field where Raman spectroscopy is one of the reference techniques^266^ that is innovating in image (see Section 4.4) and spectra processing,^267–269^ is covered by the VAMAS TWA 42. Pushed by regulatory need, many ongoing standardization initiatives on sampling and sample preparation, which are critical for data quality assurance, as well as on measurement and analytical protocols are promoted by different research groups and projects^270,271^ as well as technical committees of standardization bodies, but only a few standards exist at the moment, and these are all relatively new.^272^ Another example is in the biomedical field, where Raman spectroscopy is emerging as a promising tool for early and improved diagnosis in various tissue and biopsy samples. Traditional histopathological methods—such as the examination of H&E or immunohistochemically stained tissue sections—provide valuable clinical insights but require expert interpretation, time-consuming processing, and expensive reagents. In contrast, Raman spectroscopy offers detailed information about the molecular composition of tissues, although its clinical use is not yet widespread, partially due to the variability among different Raman instruments. Although several statistical models have been proposed to mitigate these issues, many of these studies have involved only a small number of patients and samples. As noted by C. Krafft and J. Popp in 2019, larger studies utilizing high-throughput instruments are essential to gather extensive data and develop robust classification models.^273^ Here, the Raman4Clinics COST action^274^ played a key role in highlighting the need for standardization in Raman spectroscopy for biomedical applications. As part of its efforts, it organized two large-scale interlaboratory studies (ILS) to evaluate the comparability and reproducibility of Raman-based measurements across multiple institutions. The first ILS, involving 35 Raman instruments across 15 institutions, demonstrated that standardized protocols significantly improve data reproducibility despite instrumental differences.^275^ The second ILS focused on quantitative SERS, showing that harmonized methodologies are crucial for achieving reliable and reproducible results. These studies encouraged manufacturers to include default calibration modules with transparent technical details and provide access to raw, unprocessed data, so that researchers can work with measurements that closely represent the underlying “ground truth”, while encouraging researchers to openly share data, since it becomes critical for training future statistical models. Additional research^276^ demonstrated that applying correction algorithms—such as wavenumber shift adjustments, instrument response corrections, and baseline removal—can successfully distinguish between adenocarcinoma and noncancerous intraepithelial metaplasia in esophageal tissue, even when using data from three different instruments in three different locations. More recently, Blake et el.^277^ explored esophageal tissue classification using modern machine learning techniques in a multicenter study. They found that when the same model of spectrometer and a common protocol are used across different centers, consistent data transfer can be achieved without the need for further computational corrections. These studies underscored the necessity of collaborative efforts to establish standardized Raman spectroscopy workflows.^278^

In summary, greater depth and breadth in the collaboration between academia, industry, and standardization bodies is essential to achieving a standardized, interoperable framework for Raman spectroscopy. However, documents published by standardization bodies are not open, and FAIR principles are not contemplated.

### 6.2 Raman Spectroscopy Ontology

The semantic foundation for standardizing Raman data and metadata should be built upon FAIR web ontologies. These ontologies provide a machine-readable, formally specified conceptualization of a domain by capturing the technical terms and relationships that are commonly understood and agreed upon by domain experts.^279^ This approach links a specific term from a domain’s technical vocabulary to a unique, machine-readable concept within an ontology, thereby ensuring that everyone using that term is referring to the exact same thing. Better interoperability of (meta)­data schemas can be achieved by grounding the meaning of their elements through mapping them onto terms from established ontologies. For this, such ontologies must be findable through persistent identifiers, accessible via standard protocols, interoperable through careful alignment with existing semantic standards, and reusable across different contexts and applications.

There already exist a few ontologies which cover in different degrees the Raman spectroscopy domain. The Chemical Methods Ontology (CHMO)^280^ defines classes to represent Raman microscopy, Raman spectroscopy, Raman spectrum, Raman spectrometer, and Raman microscope. The Raman spectroscopy branch contains subclasses for the most widely used Raman techniques and CHMO can be reused as a module along with other open ontologies adhering to the Open Biomedical Ontologies (OBO) Foundry principles.^281^ However, its current scope and axiomatization shows gaps and is partially confusing to domain experts with regard to using it for more fine-grained data set annotation from a research data management perspective.^282^ The Allotrope Foundation Ontology Suite (AFO)^283^ closes some of the most important gaps, such as defining classes for Raman spectrometer configuration, Raman wavenumber shift, or Raman spectroscopy parameter. However, AFO is not well suited to be combined in a modular fashion with other open ontologies, as its midlevel ontological commitment and design patterns—the chosen domain-agnostic formal modeling of the world—differs in certain key areas.^282^ Unfortunately, the documentation of AFO is still too sparse for those who are not part of the Allotrope Foundation, which makes its adoption outside of the industry rather difficult and will depend on available mappings to more open ontologies. Lastly, the Physico-Chemical Process Ontology (REX)^284^ and the Ontology of Physico-chemical methods and properties (FIX),^285^ also contain a few classes from the Raman spectroscopy domain. Yet since they are not being actively maintained anymore, they are considered defunct and should thus be seen as informative predecessors of CHMO and AFO. With the Vibrational Spectroscopy Ontology (VIBSO)^286,287^ the NFDI4Chem infrastructure project aims to extend CHMO as a domain-specific module. To achieve this goal, NFDI4Chem collaborates openly with domain experts and semantic engineers from the CHARISMA project, learned societies like IUPAC and Leibniz Institute of Photonic Technology (IPHT), the OBO Foundry and National Research Data Infrastructure (NFDI) community as well as from BASF. Although still in its early stages of development, VIBSO demonstrates how domain-specific semantic standards can be developed through community-driven processes. While its scope will also encompass the broader domain of vibrational spectroscopy, including infrared spectroscopy, VIBSO’s initial development focuses specifically on Raman spectroscopy. This focused approach enables the careful development and validation of ontology design patterns that can later be extended to other vibrational spectroscopy techniques, ensuring that the semantic foundation remains consistent across the broader field. Adhering to the OBO Foundry principles,^288^ VIBSO reuses terms and design patterns from established ontologies, most importantly the Basic Formal Ontology (BFO),^289^ the Relation Ontology (RO),^290^ the Information Artifact Ontology (IAO)^291^ and the Ontology for Biomedical Investigations (OBI),^292^ instead of redefining fundamental concepts and relations anew. This strategic decision ensures logical consistency with other scientific domains while adhering to well-defined standards and best practices for ontology development. VIBSO extends the CHMO by introducing improved axiomatization for Raman spectroscopy assays, making implicit knowledge explicit through formal logical definitions. This approach uses reasoning to account for different classification aspects such as physical effects, experimental setups, and measurement objectives. The ontology design patterns being developed in VIBSO, particularly those building upon the OBI assay pattern (see Figure 11), are intentionally designed with sufficient generality to support future expansion while maintaining semantic precision.

**Figure 11:**
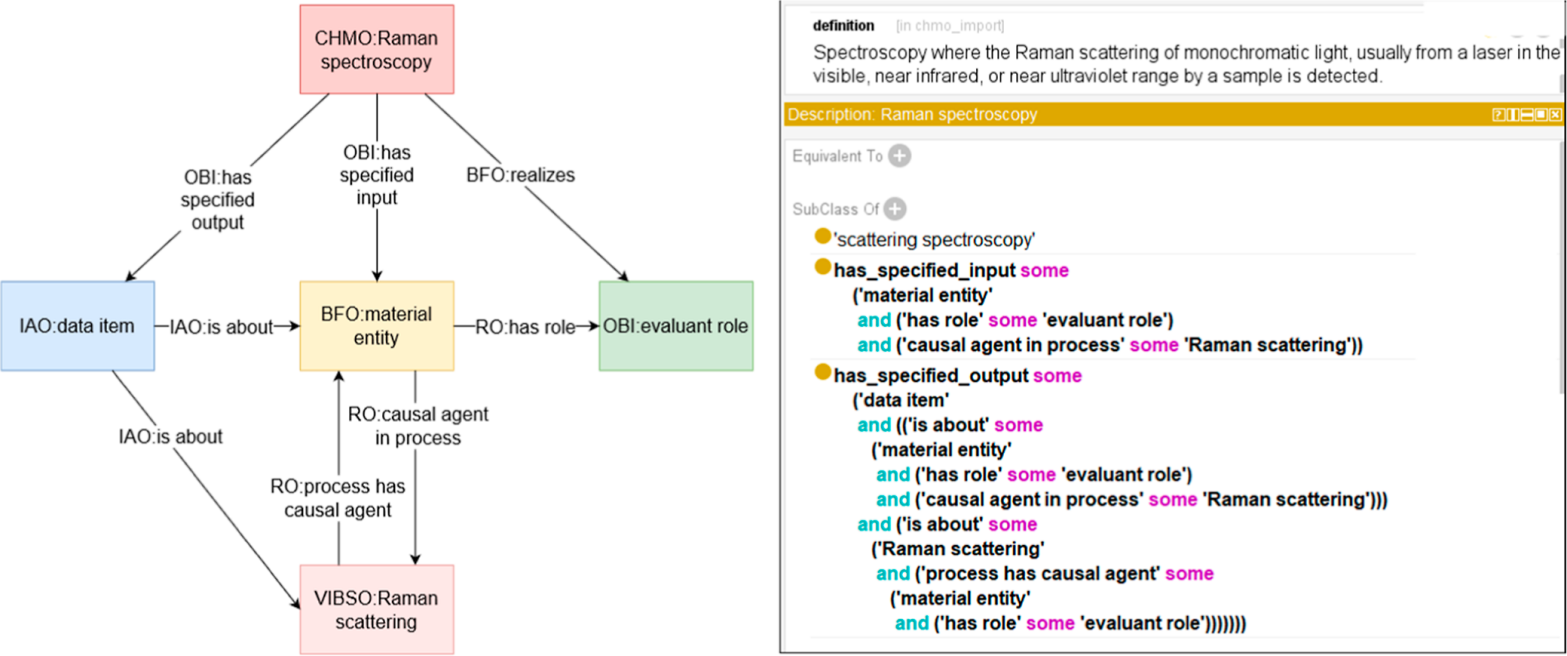
A graphical representation of VIBSO’s proposed Raman spectroscopy ontology design pattern (left) and a screenshot of its implementation in the ontology editor Protégé (right) which depict how VIBSO improves the axiomatization of CHMO by formally defining its Raman spectroscopy class according to the OBI assay pattern as a planned process that produces data about a material entity, the evaluated sample that caused a Raman scattering, as well as about said scattering effect. This design pattern also shows how VIBSO reuses terms from RO and IAO to extend OBI’s assay pattern, which already depends on terms from BFO and IAO.

The development practices behind VIBSO exemplify open science principles, with the ontology being hosted on GitHub and evolved through open development calls that welcome community contributions via issue reporting and pull requests. The project leverages the Ontology Development Kit (ODK),^293^ incorporating automated workflows and continuous integration practices that ensure sustainable maintenance and evolution. By providing this semantic foundation, VIBSO enables both immediate practical benefits in data management and long-term strategic advantages for the field. Its standardized terminology and formal definitions support the development of more sophisticated analytical tools and facilitate the creation of large, high-quality data sets needed for advanced AI applications. When combined with standardized data formats and well-designed databases, this semantic framework represents a crucial step toward achieving automated, intelligent, and scalable workflows in Raman spectroscopy while ensuring reproducibility and reliability across different experimental contexts.

### 6.3 FAIR Raman Data Structures

A FAIR data structure enables us to describe the full range of existing Raman experiments systematically, from mostly protected by intellectual property to fully open by enabling the principle of “as open as possible, as closed as necessary”.^294^ In practice, it proposes enough metadata fields to share all the information to reproduce the whole experimental setup, software, measurement and analysis protocol, while it makes mandatory only those fields required to unambiguously reproduce the shared results.

A clear definition of the data structure is necessary to interpret stored measurement data. This can range from simple human-readable, column-like, structured data with wavenumber and intensity as value pairs over to complex hierarchically structured and metadata-rich binary formats. Thereby, additional information about the devices, their settings, experimental conditions, or sample metadata is stored. This information is available to the experimentalist because it is either fixed and known from the experimental setup (lens characteristics or scattering configuration) or it is stored in (possibly multiple) output files. While such a common practice in laboratories was suitable concerning article publications, the sharing of the (raw) Raman data themselves requires a significantly larger amount of metadata to allow the unambiguous interpretation of stored experimental spectra. The goal is, thereby, to enable a set of files, in combination with a given data structure, to answer any questions of relevance that could arise during the evaluation of this experimental data by another researcher.

The data structures nowadays usually cover the most fundamental information of Raman spectra, such as the intensity distribution, wavelength used, sample type, measurement institution, publication reference, measurement date, and, usually, a limited number of other metadata fields. However, there is a lack of a well-defined vocabulary, and a variety of domain-relevant metadata fields is either not present or not required, which inherently prevents long-term reusability of the data stored within these structures. In this way, most data structures are not able to fulfill the FAIR principles.

The considerations above led to the development of the NeXus concept,^295^ with a general application definition for Raman spectroscopy called NXraman. NeXus is built on top of, e.g., the scientific open-source hierarchical data format version 5 (HDF5,^296^ see Section 6.4) and adds domain-specific rules for organizing data within HDF5 files, in addition to a dictionary of well-defined domain-specific field names. This provides a hierarchical structure with standardized metadata fields that are incorporated in the long term at NeXusformat.org. The NeXus concept allows to build upon existing definitions to extend necessary metadata fields to specialization. In this way, by a community-driven process, a specialization of NXraman could be created (e.g., with respect to time-resolved Raman spectroscopy). Additionally, other experimental techniques such as X-ray photoelectron spectroscopy, atomic force microscopy, electron microscopy, or ellipsometry can be added to extend the characterization. Furthermore, this concept allows the description of sample properties [NXsample], user metadata [NXuser] and other generic devices such as sensors [NXsensor] or positioner elements [NXpositioner]. This way, the NeXus concepts enable the description of FAIR data sets by providing a detailed and clearly defined set of (meta)­data fields with a flexibility that makes it suitable for the large heterogeneity of Raman scattering communities.

### 6.4 Raman Spectroscopy Databases and Application Programming Interfaces (APIs)

FAIR databases provide a source of spectra that can be used for spectral search, simulation development, ML model training, etc. Databases nowadays are not considered FAIR principles compliant if not offering machine readable ways to find, access, interoperate, and reuse data with none or minimal human intervention. The state-of-the-art way of building user-friendly web and mobile interfaces usually relies on backend offering API services and web browser front end, usually implemented as JavaScript client communicating with the backend, so data can be retrieved automatically from online databases using the corresponding API. The astronomy community has a long tradition of open data sharing in computer-readable form, with examples as MAST (Mikulski Archive for Space Telescopes)^297^ service API and open source packages for accessing spectroscopy data from different astronomy databases (e.g., specutils,^298^ Open Astronomy Catalog API (OACAPI),^299^ Astropy,^300^ and many others), but this is not the typical situation in Raman spectroscopy.

We have gathered in a table a comprehensive list of open databases with experimental and theoretical Raman spectra with information about their FAIRness level and, for some which do have one, the related Representational State Transfer (REST) APIs.^159^ Among them, PubChem PUG (Power User Gateway) REST API is well-known, though its main focus is on chemical compounds and biological activities, while spectra are only available as annotations (and images). The number of independently developed material databases of Raman spectra with APIs is small; an example is the Raman Open Database (ROD) REST API. A very recent development is the specification for a common REST API OPTIMADE (Open Databases Integration for Materials Design), which aims to make material databases interoperable. The OPTIMADE specification is already implemented by multiple material databases, and thus allows querying all of them in a uniform way, laying the foundation for databases federation.

The currently available open, free databases, either specific for Raman spectroscopy or for general purpose that include Raman spectroscopy data, have each from a few Raman spectra up to several thousands of data sets for materials such as organics, minerals, color pigments, archeological materials, polymers, and more. The data entries of these open databases usually contain references to publications, a type of data entry identifier, and/or references to other experimental results. There are a few metadata fields, though the level of detail is usually limited by the choice of a non-FAIR input/output file format and/or the absence of minimum requirements. While these databases fulfill the specialized needs of niche applications, only the most recent ones embrace several of the FAIR principles. Although there are no standard metrics for the evaluation of FAIR principles compliance,^301,302^ we may say that the most important requirements for a “FAIRification” of these databases would be the implementation of (i) an API for data retrieval and software integration, (ii) usage of community-defined rich metadata to enable data reproducibility, (iii) use of vocabulary to describe the metadata for interoperability. Remarkable is the Computational Raman Database, with a relevant amount of calculated spectra, more than 5000, and other related properties for a large variety of semiconductors and insulators obtained with an optimized high-throughput workflow from first principles that was validated against experimental spectra from the RUFF database.^158^

A major challenge in the development and use of Raman spectral data sets lies in concerns around data ownership and reliability. On the one hand, databases that are not rigorously curated or lack transparent provenance are distrusted by professional users, particularly in controlled environments such as accredited testing facilities or regulated industries as the pharmaceutical one. On the other hand, maintaining high-quality reference databases requires a high and sustained investment for data acquisition, curation and storage as well as software development and maintenance, including user support. As a result, many of the most trusted Raman spectral libraries are developed or maintained by commercial vendors (a relevant list can be found together with the list of open databases,^159^ see also Section 3.2 for complementary info), whose business models rely on licensing or proprietary access. This contrasts with examples from the cheminformatics field like ChEMBL and the aforementioned PubChem, both open and publicly funded. ChEMBL is a manually curated chemical database of bioactive molecules with drug inducing properties^303^ maintained by the European Molecular Biology Laboratory (EMBL) in the UK. PubChem connects chemical and clinical information with biomedical research and is maintained by the United States National Institutes of Health (NIH). In summary, while some open initiatives exist, most open Raman databases are not FAIR and limited in scope, lack robust curation, or are disconnected from regulatory workflows, so commercial Raman libraries remain dominant in applied settings due to their controlled validation pipelines and metadata completeness.

### 6.5 Data Quality

The success of AI or ML algorithms in extracting meaningful insights from Raman spectroscopy hinges on the quality and quantity of the input data for model training (see Section 5.3). Overlooking data quality can lead to spurious correlations, biased models, and ultimately, incorrect scientific conclusions. The democratisation and accessibility of the Raman technology and data, together with the necessary balance between acquisition time and spectral quality, pose a risk for data quality and call for quality assessment methods and criteria following the principles of accuracy, reliability and generalizability. We must move beyond subjective assessments and embrace quantifiable metrics across these key dimensions^304,305^ (see Figure 12).

**Accuracy and precision**: Regular instrument performance validation and calibration using well-defined standards (see Section 6.1), replicate measurements and statistical analysis of replicates should be standard practice to minimize systematic errors and the influence of outliers. For AI, inconsistent wavelength calibration introduces significant noise and hinders the model’s ability to learn true spectral features, so acceptance criteria should define the maximum allowable wavelength deviation, which should be systematically recorded and corrected. Inconsistent intensity scaling can also mislead AI algorithms relying on peak height or area for classification or regression tasks. Intensity normalization techniques (e.g., to a standard peak or total integrated intensity) are often employed, but their consistent application and documentation are critical.
**Noise and artifacts**: Low SNR obscures genuine spectral features, making it generally difficult for AI models to discern subtle differences, but for the mentioned exceptions. SNR should be quantified (e.g., using the ratio of the peak intensity to the standard deviation of the baseline^306^) and acceptance criteria should define the threshold to ensure that the algorithms are learning from actual Raman spectral features rather than random fluctuations. In addition, artifacts should be identified and either removed/corrected through appropriate processing techniques or flagged in the metadata.
**Data completeness, consistency and representativity**: Data sets must adequately represent the diversity of the samples or conditions being studied to avoid biased conclusions and ensure generalization capabilities; data augmentation techniques can sometimes help mitigate the issue of imbalanced data sets. Uniform experimental parameters and metadata are essential for AI to effectively learn relationships within a data set; the required amount of data and metadata varies significantly depending on the complexity of the problem. Missing key spectral features, sample areas, or crucial experimental details due to low spectral, spatial or temporal resolution or inadequate experimental planning and documentation can render a data set useless for certain tasks, so the minimum requirements should be defined and checked. Careful experiment design, including resolution optimization, and correlating Raman data with results from other analytical techniques.
**Credibility**: Compliance with specified formats and standards, transparency, data curation and annotation as well as validation procedures.

**Figure 12:**
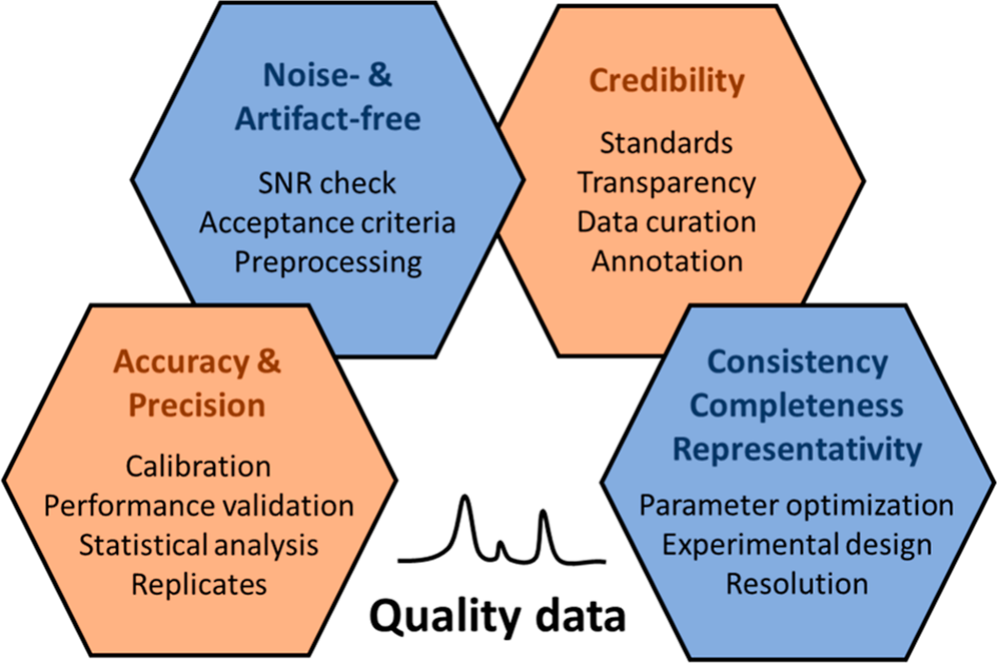
Scheme of data quality dimensions.

There are several domain-general open-source and commercial tools for data quality assurance such as Apache Griffin,^307^ Great Expectations,^308^ Informatica^309^ or Talend^310^ that provide frameworks for monitoring, validating, and documenting data quality across various stages of a data pipeline. Originally developed for general data management and big data environments, their principles could be adapted to Raman spectroscopy data sets, particularly for systematic verification of metadata integrity, file completeness, and preprocessing traceability in large or federated Raman data collections. Instead of qualitative spectral quality assessment based on visual evaluation, unbiased quantitative standardized criteria that can be applied automatically and even real time to both reject/correct low-quality spectra and optimize spectra acquisition (parameters, number of sampling points, spatio-temporal resolution, etc.) should be defined. This is, however, challenging, due to the many sources of signal variability, even if aiming at application-specific criteria.^311^ Furthermore, consistent and reliable spectra processing automation that is data-invariant and requires no user specifications has become increasingly important because processing is a rate-limiting step for applications that rely on large numbers of complex spectra. Concomitantly, automated quality control of this processing is also needed.^312^ methods to determine the quality of preprocessing include visual inspection, model performance, comparison of derivatives, and quality parameters.^312^ Butler et al. point out four key indicators of spectral quality: (i) SNR, (ii) fluorescence baseline, (iii) saturation of the detector, and (iv) photoablation.^12^ Additional causes of low quality in spectral data related to the analytical process may be errors in sample preparation, instrument calibration, processing steps, etc. For example, Tsikritsis et al.^91^ demonstrated that even manufacturer-aligned commercial Raman instruments may exhibit significant beam profile distortions due to optical aberrations, deteriorating SNR, and proposed a practical method for characterizing and optimizing beam delivery to improve measurement accuracy and reproducibility. Depending on the application, distinguishing between analytical errors (data collection and processing) and real physicochemical effects may be critical and challenging. A quantitative method to filter outliers in a data set using principal component analysis was introduced by Ramírez-Elías et al.^313^ Commercial RAMANMETRIX software offers for advanced users eight data quality filters with selectable thresholds (no criteria are defined) that can be selected as a function of the specific application to detect low quality spectra. The filters are based on selected peaks (minimal and maximal integrated normalized intensity), a reference spectrum (correlation after pretreatment/calibration or preprocessing/normalization), minimum signal-to-noise ratio, maximum background intensity, and the baseline adjusted spectra (minimum and maximum integrated intensity). Raman data can be screened for anomalies and poor-quality using quality tests.

Therefore, in the coming years, the criteria for quality must be defined in a quantifiable, traceable manner by the Raman spectroscopy community.

## 7 Conclusions and Outlook

Despite its widespread adoption across multiple disciplines, Raman spectroscopy faces significant challenges in data reproducibility, accessibility, and standardization, partly due to inconsistencies in spectral calibration, proprietary software, and limited data-sharing frameworks. To address these issues, we propose a roadmap toward a FAIR ecosystem for Raman spectroscopy, emphasizing open science principles, AI-driven optimization, and community-led standardization efforts.

A key step in this transformation would be fostering a commitment to openness and FAIR data principles to ensure Raman data is systematically structured, well-documented, and accessible to both researchers and machines. Engaging instrument manufacturers, software developers, and, in general, the private sector in this effort would accelerate adoption and ensure compatibility among evolving data-sharing frameworks and data processing tools, as well as pave the way for AI-powered new developments that may revolutionize the field.

Open Raman hardware development represents an opportunity to democratize the accessibility to Raman instrumentation, particularly through low-cost solutions and smartphone-based Raman spectroscopy. While several academic and DIY initiatives exist, a more structured effort to publish and document open-source hardware designs and control software should be encouraged, such as the OpenRAMAN project. This would facilitate the development of innovative, reproducible, tuneable, and user-friendly Raman setups for research, educational, and industrial applications.

On the software front, open-source tools are essential for advancing Raman spectroscopy. Existing libraries and platforms such as ramanchada2, RamanSPy, and ORPL are already making strides in providing accessible and reproducible spectral analysis. The integration of AI has the potential to significantly enhance Raman applications, from real-time spectral interpretation, pattern recognition, and automated feature extraction, to classification, clustering, and predictive modeling in both research and applied settings. In this regard, the Raman spectroscopy field can take advantage of AI-based developments in popular open libraries for general data processing applications. Potential applications also include the use of AI chatbots for Raman peak identification and compound recognition, as well as generative models for data augmentation or simulating spectra of molecules not yet synthesized to support virtual screening and hypothesis generation in materials science and drug discovery. Beyond data analysis, AI is also envisaged as a key tool to improve data acquisition. For example, particle recognition algorithms, which currently rely largely on image contrast, could be refined using AI-based object detection. Autofocusing systems could benefit from deep learning models trained on image or spectral feedback to optimize focus dynamically. More broadly, AI could be employed to automate and optimize acquisition parameters in real time, by continuously adjusting laser power, integration time, or scan area based on the analysis of the incoming spectral data. In summary, future AI-powered advancements, including self-calibrating instruments, real-time acquisition optimization, and fully automated processing and analysis workflows will further enhance the applicability and impact of Raman spectroscopy by enabling more efficient, adaptive, and reproducible Raman workflows. However, quality assurance as well as ethical and security considerations remain an ongoing challenge. Transparency, explainability, and reproducibility in AI-driven Raman spectroscopy could benefit not only from open-source algorithms but also from well-curated, publicly available training and validation data sets to mitigate bias and enhance model reliability. To fully realize a FAIR Raman spectroscopy ecosystem, data storage and sharing infrastructure must evolve to incorporate machine-readable and flexible data formats such as NeXus, standardized metadata and semantic frameworks such as VIBSO, and APIs for programmatic access.

Open science and FAIR principles should ideally be applied in all steps of the research and data cycle across disciplines, from the experimental design and setup to the data analysis and conclusions dissemination, including sample collection, preparation and characterization by different techniques. Instruments, samples, operators, protocols or even experiments themselves can be registered in databases that provide persistent identifiers (PIDs), e.g., ORCID for researchers, International Generic Sample Number (IGSN ID) for samples, DOI for publications, etc. These unique identifiers should be stored as part of the metadata, which allows data connection in a broad context. Research data management platforms like NOMAD can automatically resolve these PIDs to connect information from different data sets for a wider picture (See Figure 13).

**Figure 13:**
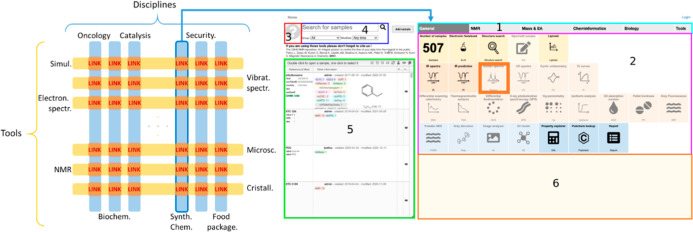
Integration of Raman spectroscopy in a broader context. Left: schematic representation of the connections between the standards or ontologies describing a theoretical or experimental method (rows) and a sample in a given discipline (columns). Right: practical example: homepage of an ELN, with tabs containing (1) type of tools, (2) available tools, (3) help, (4) sample search and group filter, (5) sample overview, (5) Information about a studied molecule linked to different characterization techniques. Adapted with authorization from Cheminfo https://docs.c6h6.org/docs/eln/uuid/158ef2f0cc85bfc5b4f2d88cff473e83.

Standardization efforts are essential to ensuring interoperability, reproducibility, and trust across laboratories and industries. Current Raman spectroscopy standards require modernization to accommodate recent advancements in instrumentation, calibration protocols, and data processing methodologies.

These developments could enable more efficient, adaptive, and reproducible Raman workflows. Ultimately, the success of this roadmap depends on interdisciplinary and international collaboration. A coordinated effort, including engagement of metrology and standardization organizations with instrument manufacturers and software developers, the academia, sectors such as forensics, security or the pharmaceutical industry, and regulatory bodies is necessary to bridge the existing gaps and establish a robust, scalable, and sustainable Raman spectroscopy ecosystem. By adopting best practices from fields like genomics, where open data and standardized databases have revolutionized research, Raman spectroscopy can achieve similar advancements in accessibility, accuracy, applicability and innovation.

This roadmap aims to lay the foundation for a modern, open, and FAIR Raman spectroscopy framework that benefits academia, industry, and society as a whole. Moving forward, continuous community engagement and technological innovation will be critical in realizing this vision.

## Supplementary Material



## Data Availability

List of Raman standards: https://doi.org/10.5281/zenodo.15086060; list of Raman databases: https://doi.org/10.5281/zenodo.15268225; list of open-source Raman spectra processing packages and libraries: https://github.com/allthingsraman.

## Notes

Table S1. Overview of the multiple beneficial effects triggered by the strategic adoption of FAIR and open science principles for optimized data-sharing in molecular sciences (PDF)

## Vocabulary

Raman spectroscopy (RS): RS is a label-free nondestructive analytical technique that uses the inelastic scattering of light to provide a “fingerprint” of a material’s chemical structure.
Surface-Enhanced Raman Spectroscopy (SERS): SERS is a technique that significantly amplifies the Raman scattering signal from molecules adsorbed on nanostructured metal surfaces, allowing for ultrasensitive detection and identification.
Tip-enhanced Raman spectroscopy (TERS): TERS is a nanoscale chemical imaging technique that combines the molecular information from RS with the high spatial resolution of scanning probe microscopy, making use of a sharp, plasmonic probe tip to enhanced electromagnetic radiation.
Stimulated Raman Scattering (SRS): SRS is a label-free spectroscopic technique that uses a pump and a Stokes beam to probe a sample’s molecular vibrations, generating a strong, amplified Raman signal when the frequency difference between the two beams matches a molecule’s vibrational frequency.
Digitalization: Digitalization is the broad process of integrating digital technologies into all aspects of a business or system, transforming how it operates, makes decisions, and interacts with customers.
FAIR principles: FAIR (Findable, Accessible, Interoperable, Reusable) principles are a set of international guidelines that guide how research data should be managed and shared with the ultimate goal to optimize the reuse of data.
