# Supplementary material for: Artificial Intelligence-Powered Raman Spectroscopy through Open Science and FAIR Principles
Source: ACS Nano. 2025 Oct 27;19(44):38189–218. doi: 10.1021/acsnano.5c09165 (PMC12613844; doi:10.1021/acsnano.5c09165)
Supplement: Supplementary file 1 [file nn5c09165_si_001.pdf]

Supporting information:

# Artificial Intelligence-Powered Raman Spectroscopy Through Open Science and FAIR Principles

Nicolas Coca-Lopez[1*], Victor Alcolea-Rodriguez[2], Miguel A. Bañares[1], Sandor Brockhauser[3], Julien Gorenflot[4], Alex Henderson[5], Ron Hildebrandt[3,12], Nina Jeliazkova[7], Nikolay Kochev[7,8], Enrique Lozano Diz[9], Zdenek Pilat[10], Dario Polli[2,11], Philip Strömert[6], Chris Sturm[12], Renzo Vanna[2], Raquel Portela[1*]

[1] Instituto de Catalisis y Petroleoquimica (ICP), CSIC, Madrid, 28049, Spain.

[2] CNR-Institute for Photonics and Nanotechnologies (CNR-IFN), P.zza Leonardo Da Vinci 32, 20133, Milan, Italy

[3] Humboldt-Universität zu Berlin, Berlin, 12489, Germany.

[4] Physical Sciences and Engineering Division (PSE), King Abdullah University of Science and Technology (KAUST), Thuwal, 23955-6900 Kingdom of Saudi Arabia

[5] Manchester Institute of Biotechnology, The University of Manchester, Manchester, M1 7DN, UK

[6] German National Library of Science and Technology (TIB) - Leibniz Information Centre for Science and Technology - University Library, Hannover, 30167, Germany.

[7] Ideaconsult Ltd., Sofia 1000, Bulgaria

[8] Department of Analytical Chemistry and Computer Chemistry, University Plovdiv, 24 Tsar Asen Str., Plovdiv 4000, Bulgaria

[9] ELODIZ Ltd, High Wycombe, Bucks, HP11 2LT, UK.

[10] Institute of Scientific Instruments of the Czech Academy of Sciences, Kralovopolska 147, Brno 612 64, Czech Republic

[11] Department of Physics, Politecnico di Milano, P.zza Leonardo da Vinci 32, 20133, Milan, Italy

[12] Felix Bloch Institute for Solid State Physics, University Leipzig, Lepzig, D04103, Germany

* Corresponding authors: raquel.portela@csic.es, nicolas.coca@csic.es

Rest of the authors contributed equally and are cited by alphabetical order

# 1 Supporting information

***Table S 1.*** *Overview of the multiple beneficial effects triggered by the strategic adoption of FAIR and open science principles for optimized data-sharing in molecular sciences.*

| **Action - trigger for the paradigm shift:** Establishing the open database of genetic sequences (GenBank®) | |
|---|---|
| **Direct effects:** | **Indirect effects:** |
| More open databases (complementary & overlapping) | Leading by example & inspiration |
| Links between databases - database networks | Increasing visibility & size of interest group |
| Standardization of data formats & annotation practices | Enabling large scale meta-analyses (& AI) |
| Development of freely available data-processing tools | Simplified handling & retrieval of specific data |
| Effective reuse of data - Improved economy of research | New therapeutics & diagnostics |
| Collaborative and interdisciplinary research | Practical impact: e.g. understanding cancer |
| Data-driven hypotheses (patterns in large datasets) | Systems biology: e.g. gene networks |
| Machine learning / AI-based tools (predictive capacity) | E.g. success predicting protein folding |
| Data sharing policies necessary | Solving authorship rights & licensing |
| Journals: data sharing improves impact of articles | Data to GenBank required for publishing |
| Funding providers: sharing data validates support for projects | Data to GenBank required for financing |
| Ethical considerations about personal (genetic) data | Guidelines for responsible data sharing |
| Bioinformatics courses (amplifying the message) | Effective training of professionals |
| Citizen science platforms (engaging public in research) | Democratize data access & demystify science |
